# Severe conservation risks of roads on apex predators

**DOI:** 10.1038/s41598-022-05294-9

**Published:** 2022-02-21

**Authors:** Itxaso Quintana, Edgar F. Cifuentes, Jeffrey A. Dunnink, María Ariza, Daniela Martínez-Medina, Felipe M. Fantacini, Bibek R. Shrestha, Freddie-Jeanne Richard

**Affiliations:** 1 Département de Biologie des Organismes et des Populations, Université de Poitiers, Poitiers, France; 2 Biodiversity and Development Institute, Unit 4, Gunner’s Park, Epping 1, Cape Town, 7460 South Africa; 3 Department of Plant Sciences, University of Cambridge Conservation Research Institute, The David Attenborough Building, Pembroke Street, Cambridge, CB2 3QZ UK; 4 Laboratorio de Ecología de Bosques Tropicales Y Primatología (LEBTYP), Universidad de los Andes, Cra 1 Nº 18A - 12, Bogotá, Colombia; 5 Panthera, 8 West 40th Str., New York, NY 10018 USA; 6 Natural History Museum, University of Oslo, Blindern, P.O. Box 1172, 0318 Oslo, Norway; 7 Fundación Reserva Natural la Palmita, Centro de Investigación, Grupo de Investigaciones Territoriales Para el Uso y Conservación de LA Biodiversidad, Bogotá, Colombia; 8 Instituto Ambiental Brüderthal, IAB, Brusque, SC 88353-190 Brazil; 9 Global Institute for Interdisciplinary Studies (GIIS), P.O. Box 3084, Kathmandu, Nepal

**Keywords:** Conservation biology, Conservation biology

## Abstract

The global expansion of road networks threatens apex predator conservation and ecosystem functioning. This occurs through wildlife-vehicle collisions, habitat loss and fragmentation, reduced genetic connectivity and increased poaching. We reviewed road impacts on 36 apex predator species and assessed their risk from current roads based on road exposure and species vulnerability. Our findings reveal all apex predators are exposed to road impacts. Eight of the ten species with the highest risk occur in Asia, although other high-risk species are present in the Americas, Africa and Europe. The sloth bear suffers the highest risk of all apex predators, followed by the tiger and dhole. Based on species risk from roads, we propose a widely applicable method to assess the potential impact of future roads on apex predators. We applied this method to proposed road developments in three areas: the Brazilian Amazon, Africa, and Nepal, to locate high-impact road segments. Roughly 500 protected areas will be intersected by these roads, threatening core apex predator habitats. We advocate the need for rigorous road development planning to apply effective mitigation measures as an urgent priority and to avoid construction in wilderness areas and predator strongholds.

## Introduction

Roads are the most widespread form of landscape modification, developed in pursuit of natural resource exploitation, agricultural, and economic development^1–2^. They commonly drive loss of ecological health and integrity^3,4^ providing human access to otherwise undisturbed areas and affecting ecosystems and wildlife^3^. Some of the impacts of roaded landscapes for wildlife are direct mortality from wildlife-vehicle collisions (WVC), land clearing driving habitat loss and fragmentation, reduced habitat quality adjacent to roads, increased access for poaching, and barriers to wildlife movement causing population fragmentation and loss of genetic connectivity^4,5^.

Roads affect almost all species groups^4^, but apex predators are acutely threatened by road development, due to their large spatial ranges, low population densities, low reproductive rates, and intolerance to increased human disturbance for most species^6^. Apex predators are defined here as non-herbivorous terrestrial mammals having an average body mass higher than 13 kg^7^, or species that are below that threshold but are main predators in their ecosystems (see Supplementary Table S1). They are simultaneously vital to the structure, functioning, and resilience of ecosystems globally^8,9^, due to their direct and indirect influence on the interactions of lower trophic levels, by displacing mesopredators and regulating large herbivore populations^8^. The maintenance of ecosystem functioning provided by apex predators is linked to a variety of ecosystem services, including carbon sequestration, water provision, and food security^9,10^. Therefore, conservation of apex predators and their habitats is exceptionally important to ensure the preservation and functionality of entire ecosystems over time^8^.

Historically, road development has been pervasive in developed countries with high Gross Domestic Product (GDP), such as Northwest Europe and the USA, as well as in more densely populated countries like India and Bangladesh^11^. Large infrastructure projects such as the Belt and Road Initiative^12^, and Africa’s Development Corridors have provided the impetus to shift this trend. Over 25 million km of newly paved roads will be constructed globally by 2050^13^. Around 90% will occur in developing nations that host critical ecosystems and rich biodiversity areas^14^ important for apex predators, such as the Amazon^15^, Africa^16^ and Southern Asia^17^. It is essential to establish a deeper understanding of the impact of current and proposed roads on apex predators. This knowledge can form the base for meaningful engagement between road planning committees, financiers, and local stakeholders to ensure biodiversity is protected while achieving the maximum social and economic benefit.

Here, we aim to assess the risk of terrestrial apex predator species from current road infrastructure and to detect impact areas of proposed road development on apex predators. We specifically: (1) conducted a systematic literature review and curated a global database of WVC to present the state of knowledge of the impact of roads on apex predators; (2) evaluated the risk to roads of 36 apex predators globally, considering the level of road exposure and the vulnerability of predators; and (3) predicted the level of impact of three future road developments by overlying the risk to roads of predators and protected areas (PAs). We used three case studies that hold critical ecosystems for apex predator diversity and where plans for road development have been proposed: the Brazilian Amazon road network expansion, development corridors in the African continent, and the completion of the Postal Highway in Nepal.

## Current knowledge of road impacts on apex predators

### Wildlife-vehicle collisions (WVC)

**Figure 1:**
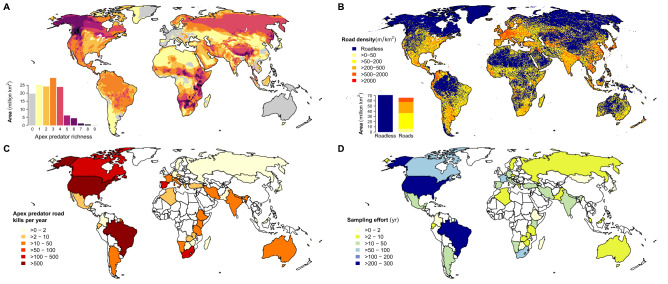
(**A**) Global apex predator richness (*n* = 36) calculated from IUCN species distribution maps^18^. (**B**) Global road density at 5-arcmin resolution, equivalent to approximately 8 km in the tropics, representing four road types (highways, primary, secondary, and tertiary roads), data from the Global Roads Inventory Project^11^. (**C**) Total number of Wildlife-Vehicle Collisions (WVC) of apex predators recorded in a single year (between 1963 and 2021) for each country with available WVC data. (**D**) WVC sampling effort per country, referred to the sum of years of study (ranging from 1963 to 2021). Maps were generated in R software v4.0.3 (https://www.R-project.org/).

The most direct impact of roads on apex predators is WVC resulting in death or injury^6^. We show evidence of almost all apex predators being impacted by WVC (Supplementary Fig. S1), with documented roadkill data for 30 of the 36 species (Supplementary Dataset 1). While it is expected that WVC are most common in areas with a combination of high predator richness (Fig. 1A) and high road density (Fig. 1B), detection is also dependent on WVC monitoring effort (Fig. 1C, D). Evidence is, thus, biased to species in countries with high road density and strong WVC monitoring effort. Indeed, such impacts were observed in Brazil, the USA, Canada, Spain and South Africa (Fig. 1). Per year, road accidents impact more than 100 individuals of American black bear (*Ursus americanus*) in the USA, maned wolf (*Chrysocyon brachyurus*), puma (*Puma concolor*) and ocelot (*Leopardus pardalis*) in Brazil, serval (*Leptailurus serval*) in South Africa, and Iberian lynx (*Lynx pardinus*) in Spain (Supplementary Fig. S1). East Africa shows a relatively high WVC incidence despite limited monitoring effort and lower density of roads, likely due to the region’s high apex predator richness (Fig. 1). Nevertheless, large gaps in documented WVC incidents exist in Southeast Asia, tropical America, and West and Central Africa (Fig. 1C). Moreover, WVC data is mainly missing for species distributed in Southeast Asia, such as clouded leopard (*Neofelis nebulosa*), Sunda clouded leopard (*Neofelis diardi*), sun bear (*Helarctos malayanus*) and Asiatic golden cat (*Catopuma temminckii*).

WVC are a particularly serious threat for apex predators as they have small population sizes frequently in contact with roads due to wide home-ranges, such as the Iberian lynx and the Asiatic cheetah (*Acinonyx jubatus venaticus*)^19,20^. In total, 229 WVC incidents involving Iberian lynx were documented between 1990 and 2020 (Supplementary Dataset 1). Likewise, for the critically endangered Asiatic cheetah, the death of one to two individuals annually (Supplementary Dataset 1) poses a significant threat to the survival of the population^20^. Factors such as road location and vehicle speed greatly influence the magnitude and severity of WVC incidents. For instance, the Iberian lynx encounters more collisions on roads bisecting its preferred habitat, and with a speed limit greater than 90 km/h^19^. Road proximity to PAs also influences collision frequency, especially when such areas maintain healthy predator populations^20^. For example, in Iran, the highest number of road collisions for the Asiatic cheetah was reported in highways crossing PAs^20^.

### Habitat loss and fragmentation

Roads are key drivers of human development and land-use conversion, causing habitat loss and degradation^3^. As roads divide habitats into progressively smaller patches, remaining natural areas become fragmented, restricting predator population connectivity^21^. Due to their need for large, undisturbed areas to support viable populations, apex predators are disproportionately affected by discontinuous habitat^6^. These effects are further intensified for species with small distributions or highly specialized habitat requirements^21^. For instance, the spectacled bear (*Tremarctos ornatus*), which inhabits the northern Andes in South America, is confined to patches of montane forest and grassland fragmented by roads^22^. Furthermore, linear infrastructures also disrupt the behaviour and distribution of prey species potentially impacting apex predators through a reverse trophic cascade in accordance with the resource dispersion hypothesis^23^.

### Genetic fragmentation

Roads drive loss of genetic diversity through direct reduction of population sizes caused by WVC mortality, and through creating physical barriers to movement, affecting population connectivity and ultimately resulting in isolation^24^. High mobility predators are particularly susceptible, as seen with bobcat (*Lynx rufus*) and coyote (*Canis latrans*) in California, and Grizzly bear (*Ursus arctos*) in western North America, which show spatial population structuration and genetic differentiation in relation to roads^25,26^. Moreover, freeways in California present complete barriers to puma movement, causing inbreeding in isolated subpopulations^27^. Likewise, populations of the ocelot in southern Texas suffer from alarmingly low genetic diversity due to extensive road development^28^. The consequences of roads in determining genetic structure are not always well documented, in part due to the relatively short period over which populations have been influenced by roads^24^. Still, a strong negative impact is expected in the future years^29,30^. Reduced genetic variability leading to a higher probability of extirpation is predicted for tiger (*Panthera tigris*) and Asiatic Black bear (*Ursus thibetanus*) populations in Asia^29,30^. Therefore, it is essential that roads do not pose absolute barriers to gene flow, allowing some individual crossing for genetic exchange^24,25^.

### Poaching and hunting

Transport infrastructure promotes human settlements and increases access to formerly remote wilderness areas, facilitating poaching for bushmeat and illegal wildlife trade^31^. Examples are found in Southeast Asia, where roads contribute to poaching in roadside forests and road networks facilitate illegal animal trade^32^. In India, poachers use rail routes to access tiger habitats^33^. The same is also reported in South America, where hunting effort is demonstrably higher near roads within PAs in the Ecuadorian Amazon^34^. Likewise, in continental Africa, snare density increases with decreasing distance from roads^35,36^. Consequently, large carnivore populations have significantly declined in heavily hunted areas^31^. In the Congo Basin, extensive bushmeat hunting led to a decline in leopards (*Panthera pardus*)^37^. Similarly, jaguar (*Panthera onca*) density decreased due to the extirpation of their prey when accessibility through roads increased in the Amazon forest^38^. The depletion of prey can also lead to human-wildlife conflicts as predators are likely to rely on livestock and anthropogenic food^39^.

## Road risk on apex predators

**Figure 2:**
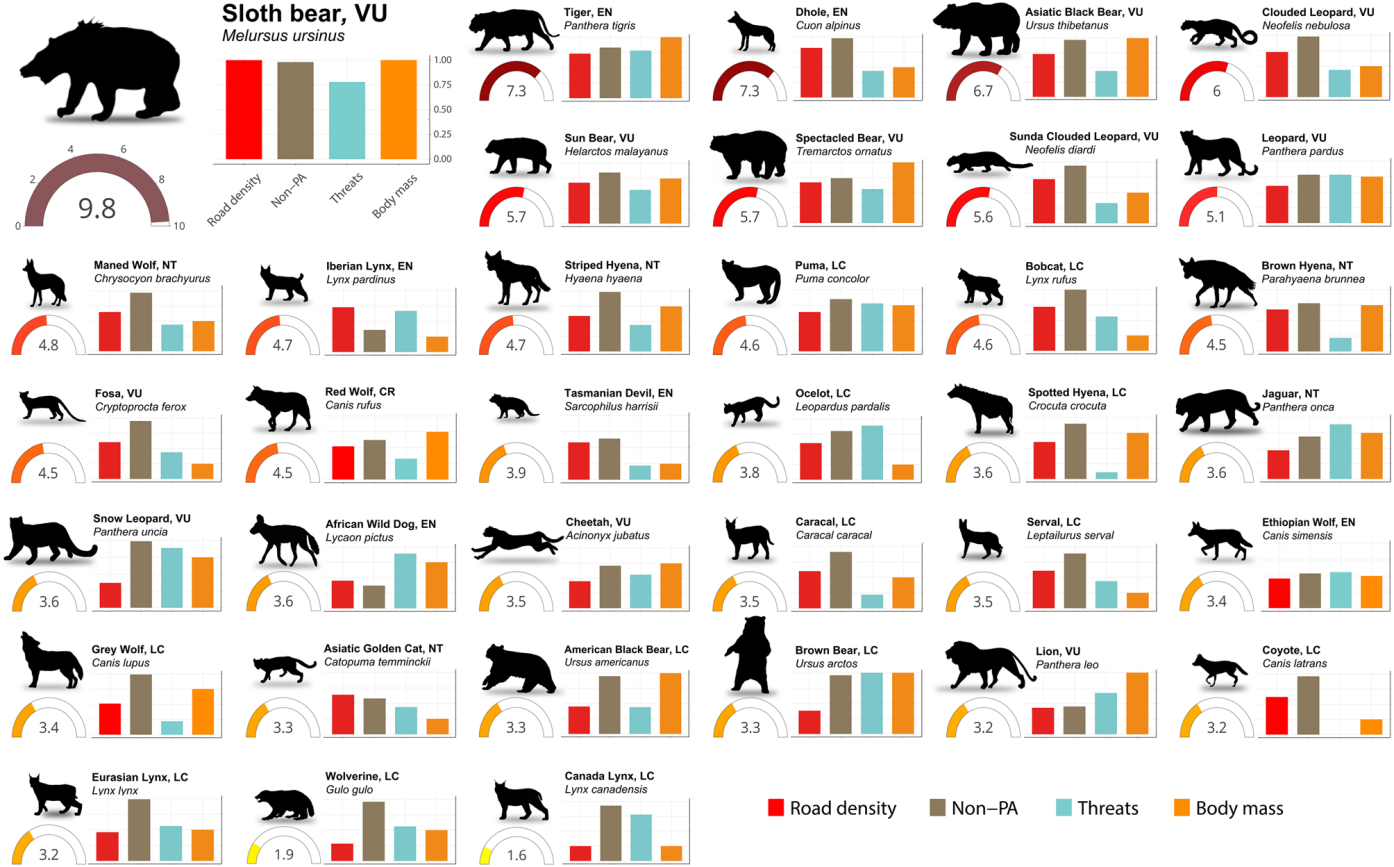
Apex predators risk to roads calculated as the product of exposure to roads (road density within the species range) and vulnerability of species. Gauges indicate the risk value of each species. Bars indicate the standardized road density, the proportion of species distribution area unprotected, standardized number of IUCN listed threats, and standardized value of the categorized average body mass (i.e., very large (> 100 kg), large (25–100 kg), medium (15–25 kg), small (8–15 kg)). The IUCN conservation of each species^18^ is shown. *P. uncia, L. pictus* and *L. canadensis* silhouettes were drawn by Gabriela Palomo-Muñoz, all predator silhouettes were acquired from PhyloPic (http://phylopic.org/).

Here we assess the risk of the current road network on apex predators based on exposure to roads and vulnerability of species (Supplementary Table S2). Our analysis highlights that all apex predator species are currently exposed to the global road network. However, the level and severity of their risk to roads varies widely (Fig. 2). With the notable exception of Iberian lynx, African wild dog (*Lycaon pictus*), lion (*Panthera leo*), and Ethiopian wolf (*Canis simensis*), more than half of the distribution of apex predator species occur outside of PAs (Fig. 2), where road development is less restricted^3,40^. Currently, the dhole (*Cuon alpinus*), tiger, Sunda clouded leopard and striped hyena (*Hyaena hyaena*) are not listed as threatened by transport infrastructures according to IUCN red list assessment^18^, however, our results clearly show that these species are among the top eight predators most at risk from roads. On the other hand, species restricted to high latitudes: Canadian lynx (*Lynx canadensis*) and wolverine (*Gulo gulo*), experience a lower risk from roads compared to other apex predators due to larger areas of natural habitat and lesser road density in boreal landscapes (Fig. 1B and Supplementary Fig. S2).

Asia is a hotspot of high-risk apex predators, hosting eight of the ten species most exposed to roads: the sloth bear (*Melursus ursinus*), tiger, dhole, Asiatic black bear, clouded leopard, sun bear, Sunda clouded leopard and leopard (Fig. 2). Indeed, the sloth bear is the most affected apex predator of all assessed species. Distributed in the Indian subcontinent, where roadless areas are almost nonexistent (Fig. 1B), the sloth bear has the highest road density among all species (303 m/km^2^ on average; Fig. 2) and almost 97% of its distribution is covered by roads (Supplementary Fig. S2). This high exposure significantly contributes to habitat fragmentation and increased mortality from vehicle collisions. From 2012 to 2017, 15 sloth bear roadkills were recorded in India (Supplementary Dataset 1); this level of mortality presents a serious threat for this species^41^. Previous studies have also highlighted the large-scale impact of roads on tiger^42^ and Asiatic black bear^43^. The dhole experiences the second highest road density (202 m/km^2^), followed by the tiger, Asiatic black bear, clouded leopard, sun bear and Sunda clouded leopard with road densities ranging from 160 m/km^2^ to 140 m/km^2^ (Supplementary Fig. S2). The latter two of these species occur in Borneo, where road density has increased due to logging for oil palm and timber plantations during the last four decades^44^.

The leopard and striped hyena also show high risk (Fig. 2). While they occur in Africa and Asia, Asian populations are more exposed to roads than those in Africa (Supplementary Fig. S2), especially in areas of South Asia where road density is extremely high (Fig. 1B). Among the species restricted to Africa, the brown hyena (*Parahyaena brunnea*) which is endemic to Southern Africa, is the predator most at risk (Fig. 2). This species is also the most exposed to roads (average road density 142 m/km^2^) mostly due to the expanded infrastructure development in South Africa (Supplementary Fig. S2). The fosa (*Cryptoprocta ferox*) follows in the level of risk (Fig. 2). Endemic to Madagascar, its habitat is heavily fragmented^45^ and 87% of its area is unprotected (Fig. 2).

In the Americas, two of the species with higher risk occur in South America: the spectacled bear and maned wolf (Fig. 2). The spectacled bear is restricted to the northern Andean mountain chain where most of the infrastructure development in western South America has occurred (Fig. 1) and as a consequence, its distribution is highly fragmented^22^. The maned wolf inhabits open habitats where agricultural expansion in conjunction with linear infrastructure has commonly occurred, therefore most of its distribution is covered by roads (average road density of 128 m/km^2^; Supplementary Fig. S2). This canid suffers excessive WVC (Supplementary Fig. S1), mostly in the Brazilian cerrado, leading to a high extinction risk of local populations^41^. Another high-risk predator in the Americas is the Puma (Fig. 2). Despite its wide distribution, from Canada to the south of the Andean mountain chain, roadless areas only occur in the Amazon basin and North America (Supplementary Fig. S2), resulting in a high average road density within its range (125 m/km^2^). Moreover, this predator has already been assessed as highly vulnerable to roads^43^.

North America and Europe host three of the most affected apex predators: bobcat, red wolf (*Canis rufus*) and Iberian lynx (Fig. 2). The bobcat occurs widely across North America^18^ and is more exposed to roads compared to red wolf (Supplementary Table S2), however, the red wolf is critically endangered due to its restricted distribution to south-eastern United States^18^. As the Iberian lynx distribution is restricted to the Iberian Peninsula, inherently its exposure to roads is very high with an average road density of 162 m/km^2^ (Supplementary Fig. S2). Most of the Iberian lynx distribution (66%) is now protected (Fig. 2) and vast conservation interventions are in place^19^, but roads still constitute an important threat to the population^43^.

Our assessment is global, therefore underestimation of road impacts on widely distributed species is likely to happen. Threats for local populations may not be immediately evident, as those reported for the jaguar in the Brazilian Atlantic forest^46^, Asiatic cheetah in Iran^20^, or Floridian subspecies of black bear (*Ursus americanus floridanus*) and Puma (*Puma concolor conyi*) which are acutely threatened by WVC (Supplementary Dataset 1). Moreover, although species such as the grey wolf (*Canis lupus*) show a lower risk to roads due to their inhospitable northern distribution, populations found in the lower latitudes still incur a high impact from roads in the form of WVC (Supplementary Fig. S1). Thus, local circumstances must be taken into consideration to assess the vulnerability of populations and to conserve species at the local and regional levels.

## Future road development case studies

**Figure 3:**
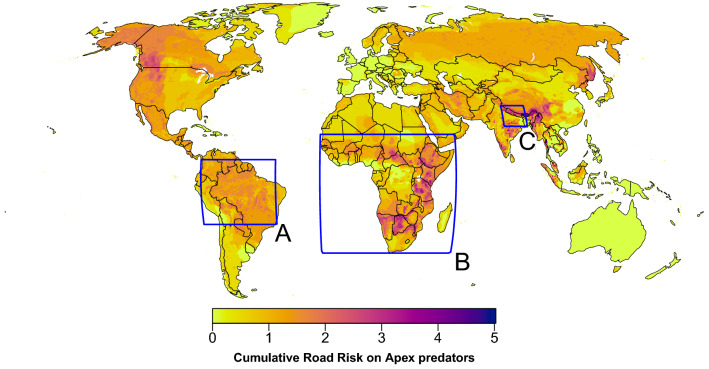
Cumulative road risk of apex predators globally used for predicting the potential impact of future roads. The cumulative risk is based on the aggregation of the road risk for each species and the presence of protected areas (see Methods), calculated at 5-arcmin resolution. Blue boxes indicate the three case studies used for assessing the potential impact of future road developments in (**A**) the Brazilian Amazon, (**B**) Africa, and (**C**) Nepal. Maps were generated in R software v4.0.3 (https://www.R-project.org/).

Here, we evaluate the potential impact of proposed road development on apex predators for each of the case studies based on the calculated cumulative road risk on apex predators (Fig. 3). Our case studies extend from specific areas to continents, where a great diversity of top predators occur: the Brazilian Amazon, the African continent, and Nepal.

### Brazilian Amazon

**Figure 4:**
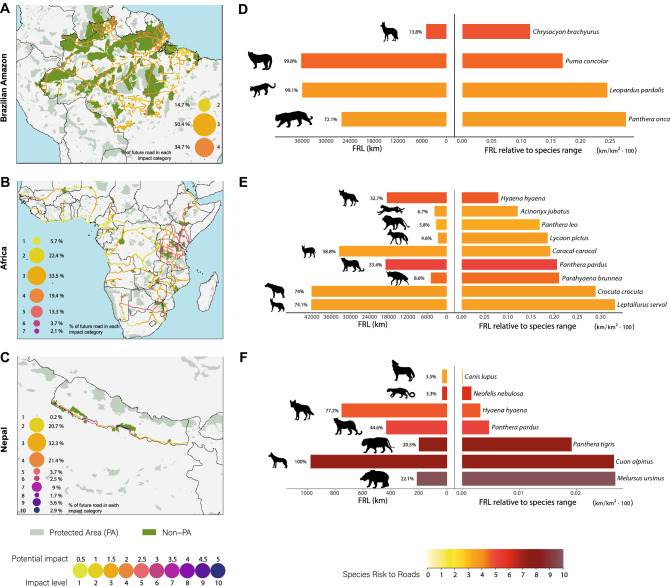
Potential impact of future road developments in (**A**) the Brazilian Amazon, (**B**) Africa, and (**C**) Nepal. Bubble sizes indicate the percentage of road length under each level of impact. Green areas denote protected areas^48^, and in darker green are the areas that will be intersected directly or within a 10-km buffer around proposed roads. Bar graphs indicate the potential impact of future road developments on predator species present in (**D**) the Brazilian Amazon, (**E**) Africa, and (**F**) Nepal. Bars on the left show the length (km) of future roads that will cross the species distribution range; numbers beside the bars indicate the percentage in relation to the complete length of the future development. Bars on the right indicate the proportion of future road length crossing the species range in relation to the size of the species total distribution. FRL = Future Road Length. Colours in bars represent the road risk for each species calculated in our assessment. Maps were generated in R software v4.0.3 (https://www.R-project.org/). *L. pictus* silhouette was drawn by Gabriela Palomo-Muñoz, all predator silhouettes were acquired from PhyloPic (http://phylopic.org/).

Due to the historical use of rivers for transport^47^, the Brazilian Amazon is currently one of the world’s largest road-free areas^11^. Road construction in the Amazon basin began in the 1950s^47^, but development has been intermittent. Proposed road expansion in the Brazilian Amazon includes roughly 16,700 km of new roads and paving a further 19,800 km of existing dirt roads (Fig. 4A). Of these, 24 projects are considered national priorities, totalling 6,032 km and mainly consisting of upgrading dirt roads^15^.

Future road development will have a high and ubiquitous impact on top predators in the Brazilian Amazon, with 85% of future roads having a level of impact between 3 and 5 (out of 10) (Fig. 4A). Although the impact may appear moderate due to the lower diversity of apex predators in the Amazon (Fig. 1A), all proposed roads will strongly affect apex predator populations across the entire Brazilian Amazon (Fig. 4A).

Almost all 36,500 km of future roads will be built inside the distribution ranges of puma (99.8% of the total road length), ocelot (99.1%) and jaguar (72.1%) (Fig. 4D). Due to their affinity for closed-canopy forests, the Amazon basin is a stronghold for the jaguar and the ocelot^18,49^. Thus, relative to the size of their respective ranges, these species will be the most impacted by the proposed roads (Fig. 4D). With large roadless areas in the Amazon, the jaguar and ocelot do not show an alarming risk from the current road network compared to other predators (Fig. 2). However, they are currently heavily impacted by WVC (Supplementary Fig. S1) and their risk to roads will likely increase with future developments. The puma will be less affected due to its wider range outside the region, but the Amazon rainforest remains critical for their conservation^18^. The maned wolf is the eighth predator most at risk from current road infrastructure (Fig. 2). However, it appears as the least impacted in our future road assessment because it inhabits open landscapes which scarcely occur in the Amazon^50^.

More than one third (38.7%) of the roads will be built inside 175 PAs, including indigenous lands and sustainable use reserves (Fig. 4A). The Yanomami indigenous territory will be significantly impacted with 1,620 km of roads crossing it. More than 700 km of new roads will bisect the largest national park in Brazil –Tumucumaque Mountains– and Raposa Serra do Sol indigenous territory (Supplementary Dataset 2). When overlaying a 10-km buffer onto the roads, a total of 321 PAs will be impacted, including a few in neighbouring countries, such as French Guiana, Venezuela, Peru and Bolivia (Fig. 4A and Supplementary Dataset 2). Roads crossing or adjacent to PAs and indigenous territories may undermine their intended protection^15^ and weaken or displace indigenous communities^51^.

Considering that most of the deforestation in the Amazon occurs adjacent to roads^47^, future developments will promote rapid and widespread forest loss^3^. Just the improvement of 2234 km of the trans-Amazonian highway may cause 561,000 ha of forest cover loss by 2030^15^. Additionally, the construction or improvement of primary roads may facilitate the development of new illegal routes^15^, expanding deforestation frontiers^3^. Habitat loss and fragmentation in the Amazon will largely impact apex predator populations, which are particularly sensitive as they require large home ranges^52^. Future road developments in the rainforest may increase apex predator mortality, as seen in the Brazilian Atlantic forests, where fragmented jaguar populations suffer high road mortality from WVC^46^. Additionally, the opening of new roads will likely increase hunting pressure by making vast areas of forest accessible^31^. Moreover, demand for tiger substitutes in traditional Chinese Medicine is promoting poaching of jaguars, pumas and ocelots^53^. Thus, the expansion of roads will imperil the conservation of forest apex predators and their remaining wilderness habitat in the Amazon basin.

### Africa

Road construction is occurring at an unprecedented rate in Africa^2,13,16^. The African Union’s Programme for Infrastructure Development proposed development corridors traversing the continent at a combined length of 56,000 km (Fig. 3B). The goal of this development is to push investment and unlock the potential of natural resources for national economies^2^. Roads, railways, powerlines, and pipelines are currently at various stages of planning and construction^16^.

Our assessment shows that the level of impact of these corridors on apex predators varies greatly across Africa (Fig. 4B). The impact from 19% of the corridors will be high (Impact level 5–7), primarily concentrated in large areas of East Africa (predominantly Tanzania, Kenya and Ethiopia), southern Africa (especially in Botswana, north-eastern South Africa, and southern Zimbabwe), as well as in road fragments in Cameroon and Senegal. Further, more than half of the development corridors (52.9%) distributed across the continent will have strong impacts on apex predators (Impact level 3–4; Fig. 4B).

Development corridors will fragment the distributions of nine African apex predator species (Fig. 4E). Around 74% of the proposed corridors will be built within the range of the serval and spotted hyena (*Crocuta crocuta*), and almost 59% in the range of the caracal (*Caracal caracal*). These three predators have the largest distributions in Africa^18^. When assessing the impacts of future roads relative to species’ distribution, the serval and spotted hyena are the most affected (Fig. 4E). Even though their risk to roads is currently moderate (Fig. 2), WVC are already affecting the species (Supplementary Fig. S1) and their exposure to roads is expected to increase in the future. Less than 10% of future roads will cross the known range of the brown hyena, but the development will cause a major impact on this species (Fig. 4E) due to its confined distribution in southern Africa^18^. The leopard will also be highly impacted by the development corridors relative to its distribution (Fig. 4E). Brown hyenas and leopards are already at high risk from roads (Fig. 2), making them particularly vulnerable to any new road development.

A total of 317 PAs will be bisected by 12.1% of the development corridors, and 1093 will be impacted within a 10-km buffer of the roads (Fig. 4A and Supplementary Dataset 2). Large wilderness landscapes and PAs will be affected. For example, the largest Ramsar wetland of international importance—Ngiri-Tumba-Maindome—will be impacted by 440 km of new roads. Located in the Congo basin, this wetland provides important forest habitat for the leopard^54^. Across East Africa, a significant number of PAs (80 in Tanzania and 49 in Kenya) will be bisected, including essential areas for the conservation of apex predators, such as the Serengeti and Katavi National Parks in Tanzania, and Tsavo National Parks in Kenya (Supplementary Dataset 2). Bisecting the Serengeti will cause devastating consequences impacting one of the world’s greatest animal migrations and causing a domino effect on healthy apex predator populations^55^.

In East and southern Africa, roads will cause severe negative impacts in areas of extremely rich apex predator diversity (Fig. 1A). The vulnerable lion, cheetah, and leopard, and the endangered African wild dog occur in isolated subpopulations across the continent. Critically important sub-populations of these species occur in Tanzania, Kenya, Botswana, Namibia, and north-eastern South Africa^18,56^ and are expected to be heavily impacted by the proposed development corridors. Additionally, Southern Africa is a stronghold for the endemic brown hyena^18^﻿. Worryingly, development corridors in Senegal and Cameroon will cross some of the last remaining habitats of west African apex predators, where lion, leopard, and African wild dog occur in isolated populations mostly confined to fragmented PAs^18^.

The development corridors may lead to new human settlements and land use transformation^16^. Corridors crossing the Congo basin could increase logging^3^ thereby impacting the leopard population. With new roads, encroachment nearby PAs will likely increase and facilitate a rise in poaching^35^. Several apex predator species suffer from WVC across East and southern Africa (Fig. 1C), and this mortality is expected to increase with new road developments. This is particularly concerning for the serval which already incurs the third highest incidence of WVC in a single year (195 individuals; Supplementary Fig. S1). Overall, poorly planned roads will intensify the threats faced by healthy populations of apex predators across Africa.

### Nepal

Nepal aims to expand its road network by completing the Postal highway. The construction of the 1792 km road traversing the southern lowlands has been delayed, with only 270 km completed so far^57^. Our analysis included only the east-west primary road of roughly 1000 km^17^ (Fig. 4C) and not the adjoining roads that make up the entire Postal Highway project.

Our analysis indicates that the planned highway will cause severe impacts on apex predators in Nepal, mostly along the central and western Terai lowlands (Fig. 4C). Segments of the future highway crossing the central Chitwan-Parsa PA complex and the western Suklaphanta National Park show an alarming level of impact in PAs with high apex predator richness (Impact level 10). Across the west Terai, almost 20% of the road to be built will cause acute impacts on apex predator populations with impact levels ranging from 6 to 9. On the other hand, in the eastern Terai, the impact will be moderate as 20.9% of the proposed road length shows impact levels ranging from 1 to 2 (Fig. 4C), due to the lower apex predator richness.

The Postal highway will cross the distribution area of seven apex predator species (Fig. 4F). There are, however, no recent records of the grey wolf in the Nepalese lowlands despite being considered part of the species’ global distribution^58^. The new highway will be entirely built within the range of the dhole. Additionally, 77% and 45% of the total length will cross the distribution of the striped hyena and leopard respectively (Fig. 4F), species currently at high risk from roads (Fig. 2). Other impacted species include sloth bear, dhole and tiger, which are also found to be the three species most at risk from current roads (Fig. 2). Additionally, relative to their respective species distributions, these vulnerable apex predators will be severely affected by the planned road (Fig. 4F).

If built as planned, 16.5% of the highway’s length will intersect a proposed conservation area and four National Parks including their adjacent buffer zones. Adding a 10-km buffer, the highway is expected to impact eight PAs in Nepal and five transboundary PAs from India (Fig. 4C and Supplementary Dataset 2). In total, roughly 97 km of the highway will cross the Chitwan-Parsa complex. The Chitwan National Park is an important conservation area and a UNESCO World Heritage Site where six apex predators, expected to be affected by future roads (Fig. 4F), co-occur^58^. However, the supreme court of Nepal has ordered a halt to the construction of a new road in the Chitwan National Park until environmental impact assessments are approved by the National Park, UNESCO and other stakeholders^59^. The second most impacted PA is Suklaphanta National Park and its buffer zones –key areas for tiger conservation^58^– where almost 26 km of the highway is planned to be built (Supplementary Dataset 2).

These PAs and adjacent wildlife corridors are part of the Terai Arc Landscape (TAL), a conservation initiative established to bridge PAs and habitat corridors allowing the connectivity of large-mammal metapopulations^60^. The TAL extends along the south-western region of Nepal being a stronghold for both the sloth bear and tiger population in Nepal^18,58,60^. Worryingly, it is expected to incur severe impacts from the proposed highway. The existing east-west Mahendra highway (running parallel to the proposed Postal highway) has already brought negative outcomes to the TAL through habitat fragmentation and quality reduction, obstruction of wildlife movements^61^ and increased incidence of WVC with tigers, leopards and striped hyenas (Supplementary Dataset 1). If not planned properly, the proposed Postal highway will degrade remaining habitats, jeopardise the connectivity of the TAL, and increase the threats faced by important apex predator populations.

## Final remarks and conclusions

We reviewed how existing road networks produce substantial impacts on apex predators. We developed a method to assess the risk of predators from roads globally and showed that all species are currently exposed to the road network, most notably in Asia. Regionally, species are critically impacted by direct mortality but the impact of WVC is difficult to assess at the global scale due to the depauperate data in many countries and the underestimation of roadkills. Furthermore, the expected rapid rise of road development in developing countries will intensify the risk of apex predators and their habitats. We proposed a widely applicable method to estimate the potential impact of future road development on apex predators and applied it to three case studies. This method can be used to assess the potential impact of new roads at the country, regional or continental scale. We identified road segments in Nepal and Africa expected to cause the most severe impact crossing distributions of numerous apex predators. We also showed that proposed roads will impact forest predators over the entire Amazon basin (the largest road-free area of tropical rainforest) where deforestation and poaching are prone to increase with new developments. Roughly 500 PAs will be intersected by roads across our three case studies, threatening core habitats for apex predators. The impact of roads extends beyond predators to their habitats, potentially disrupting ecosystem functioning and stability^8,9^. By indicating species at risk from roads and future road segments with a high potential impact, our spatial analysis identifies key regions for the implementation of protective measures and the conservation of apex predators.

To reduce the environmental impact of infrastructure developments, the mitigation hierarchy defines a four-phase process: the first and most important step is avoidance, followed by minimization, rehabilitation, and biodiversity offsetting^62,63^. In accordance with the mitigation hierarchy, our analysis supports the calls made elsewhere: existing intact areas, such as pristine regions of the Amazon rainforest and African wilderness areas, should remain road-free^1,14,40^. All proposed road developments should also expressly avoid PAs^1,14,40^. New roads in these regions will prompt habitat clearing and overexploitation of wildlife^1,3^. Future roads should simultaneously avoid rich areas of co-occurring apex predators and areas with low richness where ecosystem function is dependent on one apex predator species. When road construction is unavoidable, the full suite of mitigation measures must be implemented with long-term monitoring to assess their effectiveness. The combination of crossing structures (over- and under-passes) and roadside fencing is the best known measure to minimize the impact on apex predators through reducing mortality and maintaining connectivity^6^. Particularly, when located in WVC hotspots and high-quality species habitat^64^. Finally, biodiversity offsetting should only be used as a last resort to complement the initial three phases of the mitigation hierarchy^62,63^, and not to justify road development.

It is vital that road planning committees include conservation scientists, the voices of all stakeholders –from local communities to government officials– and even wildlife protection organisations. Similarly, development financiers need to better integrate the mitigation hierarchy into their funding agreements, ensuring that the ultimate objective of no net biodiversity loss is maintained throughout^62,63^.

Our findings highlight the current risk of apex predators and future negative impacts from roads, assessed with methods that can be extrapolated to other species groups or areas of interest. We advocate the need to avoid the construction of environmentally harmful roads in wilderness areas, better road development planning and the installation of effective mitigation measures to protect apex predators, their habitats and ultimately ecosystem functioning.

## Methods

### Selection of apex predator species

Apex predators are defined here as terrestrial mammalian carnivores with an upper limit body mass (ULBM) of 18–34 kg or an average body mass (ABM) above 13 kg^7^. Species below this threshold that were previously found to occupy high trophic levels while exerting top-down regulation on ecosystem functioning were also included. A total of 36 species were considered (Supplementary Table S1) for which an IUCN range map is available^18^.

### Wildlife Vehicle Collision data collection

To collate published Wildlife-Vehicle Collision records (Supplementary Fig. S1), systematic searches were conducted in Web of Science, SCOPUS, Crossref and Google scholar, in English, Spanish, Portuguese and French, using the following queries: (genus NEAR species) AND (road NEAR impact) OR (road NEAR kill) OR (wildlife NEAR vehicle NEAR collision); (atropellamientos OR colisión) AND (fauna OR animales) AND/OR carnívoros; atropelamentos AND (animais AND/OR silvestres) AND (carnivora OR predadores de grande porte); (collisions NEAR Faune AND/OR véhicules) AND/OR carnivore; respectively. Data from articles, congress proceedings, book chapters, databases, reports, news and thesis were included. We found 190 documents presenting WVC records, ranging from 1963 to October 2021. The number of killed individuals and study length (*i.e.*, years of study) were compiled; missing data values either for the number of individuals or years of study were assumed to be one. The rate of killed individuals per year was calculated as the fraction of total killed individuals over study length.

### Underlying spatial data

Current global road density raster files at a spatial resolution of 5-arcminutes were downloaded from Globio^11^. Road types 1 to 4 (*i.e.*, highways, primary, secondary and tertiary roads) were aggregated to estimate the total road density in m/km^2^. Road type 5 (*i.e.*, local roads) were excluded due to spatial bias towards developed countries and mainly limited to large urban areas^11^. Georeferenced vector data for IUCN extant apex predator global distributions^18^ and PAs from the Word Database on Protected Areas (WDPA)^48^ were rasterized and assigned binary values for presence and absence using the same spatial resolution of road density data (*i.e.*, 5-arcmin, equivalent to ~ 8 km in the tropics). Apex predator species richness was calculated by overlaying all species distribution raster layers. All analyses and visual outputs were conducted and processed in R software^65^.

### Species Risk to Roads

The risk to roads of each apex predator species *i* was calculated as the product of likelihood (*i.e.*, exposure to roads) and severity (*i.e.*, species vulnerability):

1 $$Road\,Risk_{i} = Road\,exposure_{i}^{*} \cdot Vulnerability_{i}^{*}$$

Road exposure is the square root of the density of roads within the range of a species *i*, which is calculated as the total length of roads inside the predator distribution (in m) divided by the area of its distribution (in km^﻿2^﻿):

2 $$Road\,exposure_{i} = \sqrt {Road\,density}_{i}$$

Vulnerability for a species *i* is the aggregation of the proportion of range outside protected areas ($nPA_{i}^{*}$), the conservation status value ($IUCN_{i}^{*}$) categorized from Least Concern (LC = 1) to Critically Endangered (CR = 5), the number of threats as listed by the IUCN^18^ ($TH_{i}^{*}$) and the average body mass ($BM_{i}^{*}$) categorized as: very large predators (> 100 kg = 4), large predators (25–100 kg = 3), medium predators (15–25 kg = 2), small predators (8–15 kg = 1) (Supplementary Table S1). Body mass was interpreted as a proxy for home range size^66^, therefore bigger apex predators have an increased likelihood of encountering a road as a consequence of their large home ranges^6^, however, we clarify that this is a general rule as there are small-sized mammals with wide home ranges (*e.g.*, the wolverine). Weighting coefficients were assigned to variables based on their relative importance. We considered non-protected distribution area (*nPA*) and IUCN category (*IUCN*) most important for assessing species vulnerability due to the crucial role PAs play in carnivore species conservation^67^ and the importance of IUCN red list classification for assessing species conservation needs^68^. Number of threats (*TH*) and body mass (*BM*) were given half the weight because they do not strictly define how vulnerable a species is.

3 $$Vulnerability_{i} = nPA_{i}^{*} + IUCN_{i}^{*} + 0.5 \cdot TH_{i}^{*} + 0.5 \cdot BM_{i}^{*}$$

In Eqs. (1) and (3) the asterisk (*) denotes that the variable was previously normalised for scale uniformity by dividing by its maximum value.

### Impact assessment of future road developments

To evaluate the potential impact of future roads on apex predators, the species risk to roads (as calculated in the previous section) and protected areas were spatially overlaid using raster data at 5-arcmin resolution, equivalent to ~ 8 km in the tropics. First, risk values were assigned to pixels corresponding to the distribution of the species, after which all species layers were overlaid to form the cumulative risk. Additionally, pixels corresponding to protected areas were given the average risk value of all species present in that pixel, according to the following formula:

4 $$Cumulative\,Road\,Risk_{px} = SSR_{px} + \beta \cdot PA_{px}$$

where for each pixel *px*, $SSR_{px}$ = sum of the species risk value for all species present in the pixel, *β* = average value of species risk for the pixel, and $PA_{px}$ = protected area expressed as binary values (0,1). Therefore, the value of each pixel corresponds to the sum of the risks of all species present in that pixel adding the average of the risks if protected (see Fig. 3).

The cumulative road risk of apex predators allows us to obtain the potential impact of future roads. To assess the impact of future developments we selected three case study areas of varying spatial scales based on their importance for apex predator species conservation and the availability of spatial data on proposed road developments. The Brazilian Amazon is currently the largest road-free area^11^ and an important stronghold for forest apex predator populations^18^. The continent of Africa supports a great diversity of apex predators and critically, road construction is occurring at an unprecedented pace and extent^2,13,16^. Despite having a relatively small area, Nepal holds high richness and important populations of Asiatic apex predators^58^. Vector data for future road developments were collected for the three regions: Brazil^69^ masked by the amazon region^70^, African development corridors^16^ and the Postal highway in Nepal^17^. Road segments of future developments were assigned with the cumulative road risk values of the overlaying pixel. Finally, PAs that will be intersected by future road construction as well as affected by a 10-km buffer along each planned road were identified.

## Supplementary Information

Supplementary Information.

Supplementary Legends.

Supplementary Dataset S1.

Supplementary Dataset S2.

## Data Availability

The authors declare that the data supporting the findings of this study are available within the paper and its supplementary information files. All analyses were performed using publicly available datasets referenced in the Methods section of this manuscript.
