# Supplementary material for: Severe conservation risks of roads on apex predators
Source: Sci Rep. 2022 Feb 21;12:2902. doi: 10.1038/s41598-022-05294-9 (PMC8861172; doi:10.1038/s41598-022-05294-9)
Supplement: Supplementary file 1 — Supplementary Information. [file 41598_2022_5294_MOESM1_ESM.pdf]

SUPPLEMENTARY INFORMATION

Severe conservation risks of roads on apex predators

Quintana I.[1,2,*], Cifuentes E.F.[1,3,4,*], Dunnink J.A.[1,5,*], Ariza M.[1,6], Martínez-Medina D.[1,7], Fantacini F. M.[1,8], Shrestha B.R.[1,9], Richard F-J.[1]

[1]Département de Biologie des Organismes et des Populations, Université de Poitiers, Poitiers, France

[2]Biodiversity and Development Institute, Unit 4, Gunner's Park, Epping 1, Cape Town 7460, South Africa

[3]Department of Plant Sciences, University of Cambridge Conservation Research Institute, The David Attenborough Building, Pembroke Street, Cambridge CB2 3QZ, UK

[4]Laboratorio de Ecología de Bosques Tropicales y Primatología (LEBTYP), Universidad de los Andes, Cra 1 Nº 18A - 12, Bogotá, Colombia

[5]Panthera, 8 West 40th Str. New York, NY, USA. 10018

[6]Natural History Museum, University of Oslo, P.O. Box 1172 Blindern, 0318 Oslo, Norway

[7]Fundación Reserva Natural La Palmita, Centro de Investigación, Grupo de Investigaciones Territoriales para el Uso y Conservación de la Biodiversidad, Bogotá, Colombia

[8]Instituto Ambiental Brüderthal, IAB, 88353-190, Brusque/SC, Brazil

[9]Global Institute for Interdisciplinary Studies (GIIS), P.O. Box 3084, Kathmandu, Nepal

*Corresponding author. Email: itxasoqg@hotmail.com (I.Q.); ed-cifue@uniandes.edu.co (E.F.C.); jeffdunnink@gmail.com (J.D.)

All I.Q., E.F.C., J.D., M.A., D.M.M., F.M.F., B.R.S., F-J.R., conceived, designed, and wrote the manuscript. E.F.C. carried out spatial analysis and maps. I.Q. and E.F.C designed and created formulae and figures. M.A., D.M.M, F.M.F., B.R.S., I.Q., J.D., and F-J.R. collected articles and extracted data for wildlife-vehicle collision.

Supplementary Table S1. Selected apex predator species including reference and justification. Note that "transition to apex predator" refers to the study by Wallach et al.[1] in which the transition from apex- to meso-predator occurred in species with an average body mass form 13-16 kg. Species that fall over this threshold but have a diet based mainly on plants (*e.g.* Panda and civet) and marine and semi-aquatic (*e.g.* polar bear, otters and pinnipeds) were excluded. Species below this weight that were previously found to occupy high trophic levels while exerting top-down regulation on ecosystem functioning were also included. Dingo was excluded from the analysis due to the lack of an available IUCN distribution map[2]. Data for mass was obtained from Wallach et al.[1] and from Jonas et al.[3] for the Tasmanian. ULBM= Upper limit body mass, and ABM= Average body mass.

**Family**	**Species name**	**Common name**	**Justification**	**ULBM (kg)**	**ABM (kg)**
Canidae	*Canis latrans*	Coyote	Transition to apex predator[1]	34	13
Canidae	*Canis lupus*	Gray wolf	Apex predator[1]	80	27
Canidae	*Canis rufus*	Red wolf	Apex predator[1]	41	25
Canidae	*Canis simensis*	Ethiopian wolf	Transition to Apex predator[1]	20	15
Canidae	*Chrysocyon brachyurus*	Maned wolf	Apex predator[1]	25	22
Canidae	*Cuon alpinus*	Dhole	Apex predator[1]	25	18
Canidae	*Lycaon pictus*	African wild dog	Apex predator[1]	34	27
Dasyuridae	*Sarcophilus harrisii*	Tasmanian devil	Apex predator[4] despite being below ABM threshold.	NA	8.2
Eupleridae	*Cryptoprocta ferox*	Fossa	Apex predator in Madagascar (largest carnivore)[5], despite being below ABM threshold.	12	10
Felidae	*Acinonyx jubatus*	Cheetah	Apex predator[1]	65	54
Felidae	*Caracal caracal*	Caracal	Transition to apex predator[1,6]	20	16
Felidae	*Catopuma temminckii*	Asiatic golden cat	Transition to apex predator[1]	16	14
Felidae	*Leopardus pardalis*	Ocelot	Transition to apex predator[1]. Its density seems to not be affected by larger cats densities and affect the smaller ones in opposite trend[7]	20	9
Felidae	*Leptailurus serval*	Serval	Transition to apex predator[1]	19	13
Felidae	*Lynx canadensis*	Canada lynx	Apex predator[8] despite being below ABM threshold.	11	10
Felidae	*Lynx lynx*	Eurasian lynx	Apex predator[1]	38	23

Felidae	*Lynx pardinus*	Iberian lynx	Apex predator[9] despite being below ABM threshold.	15	11
Felidae	*Lynx rufus*	Bobcat	Apex predator[10] despite being below ABM threshold.	14	9
Felidae	*Neofelis diardii*	Clouded leopard	Apex predator[1]	23	20
Felidae	*Neofelis nebulosa*	Clouded leopard	Apex predator[1]	23	20
Felidae	*Panthera leo*	African lion	Apex predator[1]	272	175
Felidae	*Panthera onca*	Jaguar	Apex predator[1]	125	81
Felidae	*Panthera pardus*	Leopard	Apex predator[1]	70	54
Felidae	*Panthera tigris*	Tiger	Apex predator[1]	350	120
Felidae	*Panthera uncia*	Snow leopard	Apex predator[1]	50	50
Felidae	*Puma concolor*	Puma	Apex predator[1]	105	63
Hyaenidae	*Crocuta crocuta*	Spotted hyena	Apex predator[1]	86	63
Hyaenidae	*Hyaena hyaena*	Striped hyena	Apex predator[1]	55	40
Hyaenidae	*Parahyaena brunnea*	Brown hyena	Apex predator[1]	73	42
Mustelidae	*Gulo gulo*	Wolverine	Transition to apex predator[1]	35	16
Ursidae	*Helarctos malayanus*	Sun bear	Apex predator[1]	70	45
Ursidae	*Melursus ursinus*	Sloth bear	Apex predator[1]	190	100
Ursidae	*Tremarctos ornatus*	Spectacled bear	Apex predator[1]	200	118
Ursidae	*Ursus americanus*	American black bear	Apex predator[1]	400	154
Ursidae	*Ursus arctos*	Brown bear	Apex predator[1]	680	278
Ursidae	*Ursus thibetanus*	Asiatic black bear	Apex predator[1]	200	104

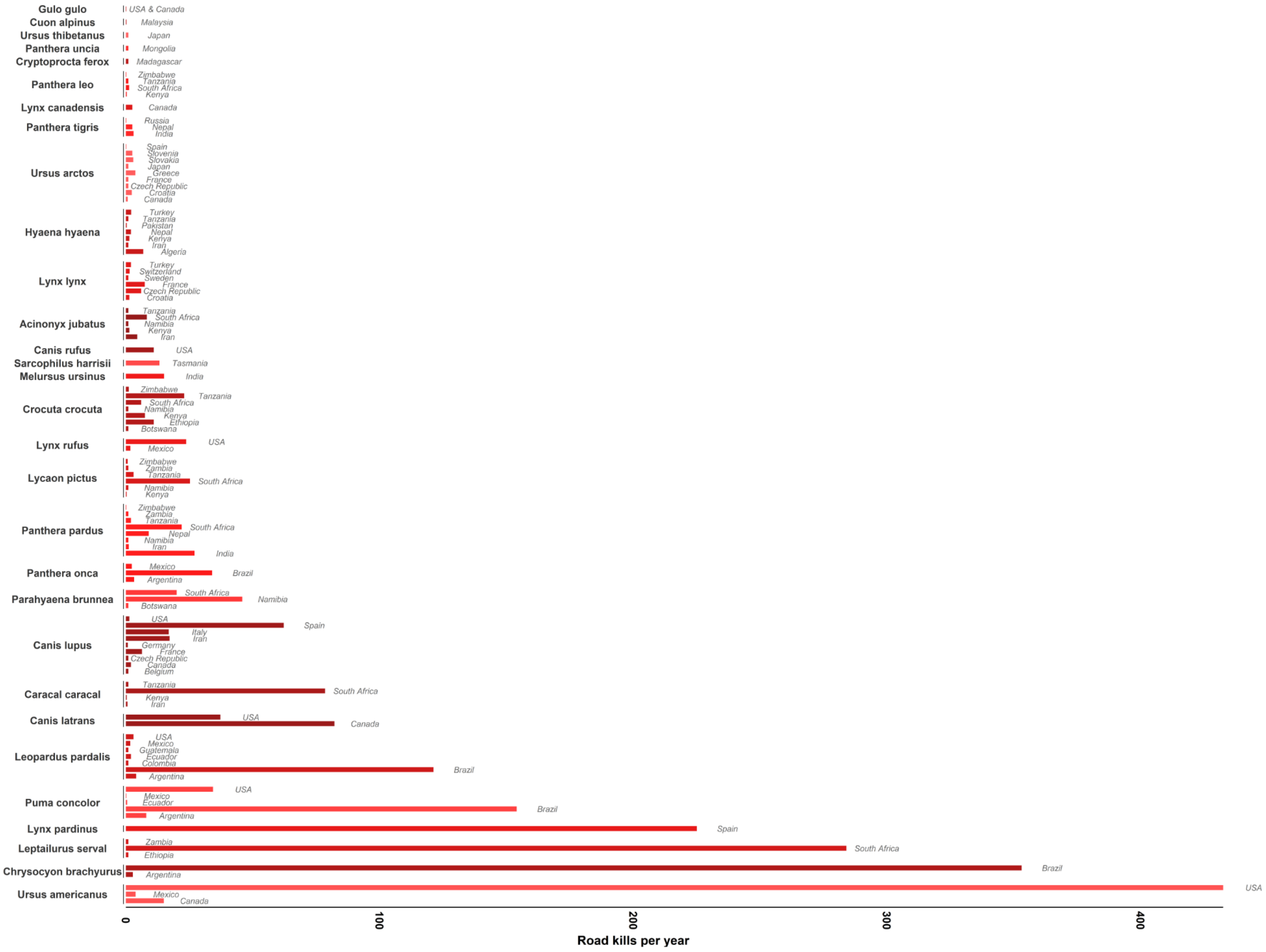

Supplementary Fig. S1. Collated wildlife-vehicle collision data from 1963 to 2021. Total number of individuals killed in a year for each species in each country is shown.

Supplementary Table S2. Variables used for the calculation of the risk to roads of each of the 36 apex predator species. Road exposure was calculated by square rooting the sum of road type 1, 2, 3 and 4 densities. Vulnerability to road was calculated by adding the normalised values of proportion of area non-protected, IUCN category, number of threats and body mass category. Proportion of area was calculated dividing the respective area by the total distribution area of each species. IUCN category was categorized as: Least Concern (LC =1), Near Threatened (NT =2), Vulnerable (VU =3), Endangered (EN =4) and Critically Endangered (CR =5). Total number of threats were calculated by adding the different types of threats listed in the IUCN. For body mass category the average body mass of each species was categorized as: Very large predators (>100 kg) =4, large predators (25-100 kg) =3, medium predators (15-25 kg) =2, small predators (8-15 kg) =1.

Species name	**Road exposure (normalised)**	Road type 1 density (m/km^2)	Road type 2 density (m/km^2)	Road type 3 density (m/km^2)	Road type 4 density (m/km^2)	Road total density (m/km^2)	**Vulnerability (normalised)**	Distribution area (km^2)	Proportion of area non protected	IUCN category	Total number of threats	Body mass category
Melursus ursinus	**1.00**	0.16	15.69	110.63	176.03	302.52	**0.98**	802298.49	0.89	3	7	4
Panthera tigris	**0.73**	0.62	12.55	52.22	97.01	162.40	**1.00**	1043977.05	0.76	4	7	4
Cuon alpinus	**0.82**	2.08	21.49	59.69	118.62	201.88	**0.89**	3666821.31	0.89	4	4	2
Ursus thibetanus	**0.73**	14.74	36.79	34.79	75.53	161.85	**0.91**	4084322.14	0.88	3	4	4
Neofelis nebulosa	**0.73**	24.86	41.48	35.45	60.23	162.02	**0.81**	2062832.44	0.89	3	4	2
Helarctos malayanus	**0.68**	0.15	12.78	15.29	113.54	141.75	**0.83**	1520003.10	0.78	3	5	3
Tremarctos ornatus	**0.67**	0.24	10.99	22.62	103.70	137.55	**0.84**	348487.90	0.67	3	5	4
Neofelis diardi	**0.72**	0.00	4.25	3.44	147.73	155.41	**0.78**	636326.99	0.86	3	3	2
Panthera pardus	**0.60**	0.67	10.10	31.05	66.10	107.92	**0.85**	9307884.16	0.71	3	7	3
Chrysocyon brachyurus	**0.65**	1.93	13.11	32.42	80.39	127.85	**0.73**	4419764.48	0.89	2	4	2
Lynx pardinus	**0.73**	43.91	7.55	43.29	67.34	162.08	**0.64**	5067.63	0.33	4	6	1
Hyaena hyaena	**0.59**	1.00	9.54	28.87	66.93	106.34	**0.79**	23741455.63	0.91	2	4	3
Puma concolor	**0.64**	2.42	17.15	36.62	68.43	124.61	**0.72**	21558871.97	0.78	1	7	3
Lynx rufus	**0.72**	9.77	32.92	92.68	21.29	156.66	**0.64**	10049581.71	0.91	1	5	1
Parahyaena brunnea	**0.68**	2.53	18.72	40.87	79.67	141.78	**0.66**	2342319.77	0.71	2	2	3
Cryptoprocta ferox	**0.60**	0.00	3.78	5.62	98.47	107.87	**0.75**	506564.31	0.87	3	4	1
Canis rufus	**0.52**	0.00	40.95	40.80	0.00	81.74	**0.86**	4796.65	0.57	5	3	3
Sarcophilus harrisii	**0.58**	0.00	20.77	16.56	65.24	102.57	**0.67**	72746.47	0.58	4	2	1

Leopardus pardalis	**0.60**	0.97	14.36	30.76	63.09	109.19	**0.62**	14816692.48	0.73	1	8	1
Crocuta crocuta	**0.60**	0.09	8.65	18.91	81.24	108.90	**0.61**	14699732.25	0.82	1	1	3
Panthera onca	**0.47**	0.21	7.36	15.37	44.76	67.70	**0.76**	9548803.68	0.63	2	8	3
Panthera uncia	**0.37**	1.05	9.16	11.09	20.61	41.92	**0.96**	3481591.63	0.91	3	8	3
Lycaon pictus	**0.45**	0.03	3.61	8.07	49.71	61.41	**0.79**	1431465.34	0.34	4	8	3
Acinonyx jubatus	**0.45**	0.06	4.96	12.98	42.93	60.93	**0.78**	3166049.42	0.65	3	5	3
Caracal caracal	**0.60**	0.98	10.68	25.07	71.83	108.55	**0.58**	17619382.46	0.83	1	2	2
Leptailurus serval	**0.61**	0.43	10.35	23.04	78.29	112.10	**0.57**	12849912.72	0.81	1	4	1
Canis simensis	**0.46**	0.00	29.73	14.14	19.66	63.54	**0.74**	13560.29	0.49	4	5	2
Canis lupus	**0.51**	4.48	13.85	27.19	31.67	77.20	**0.67**	52111415.13	0.90	1	2	3
Catopuma temminckii	**0.64**	5.45	23.97	22.74	69.96	122.12	**0.53**	606267.68	0.53	2	4	1
Ursus americanus	**0.45**	4.11	15.40	35.34	6.00	60.86	**0.74**	11091035.39	0.87	1	4	4
Ursus arctos	**0.38**	1.98	7.19	22.43	12.34	43.94	**0.86**	25554439.54	0.88	1	9	4
Panthera leo	**0.43**	0.09	4.03	11.36	40.62	56.09	**0.75**	1964996.46	0.41	3	6	4
Canis latrans	**0.62**	6.94	25.09	66.97	17.19	116.19	**0.51**	17300007.48	0.88	1	0	1
Lynx lynx	**0.46**	2.51	10.15	32.46	20.27	65.39	**0.68**	21017161.31	0.90	1	5	2
Gulo gulo	**0.28**	0.76	3.37	14.46	4.84	23.43	**0.67**	24756814.38	0.87	1	5	2
Lynx canadensis	**0.25**	0.74	7.81	6.39	3.77	18.71	**0.65**	8317875.83	0.84	1	7	1

Supplementary Fig. S2. Road density within each apex predator species distribution representing four road types (highways, primary, secondary and tertiary roads) at 5 arcmin resolution (approx. 8 km in the tropics). Road density data obtained from Global Roads Inventory Project[11]. Maps were generated in R software v4.0.3 (https://www.R-project.org/). Find maps in the following pages.

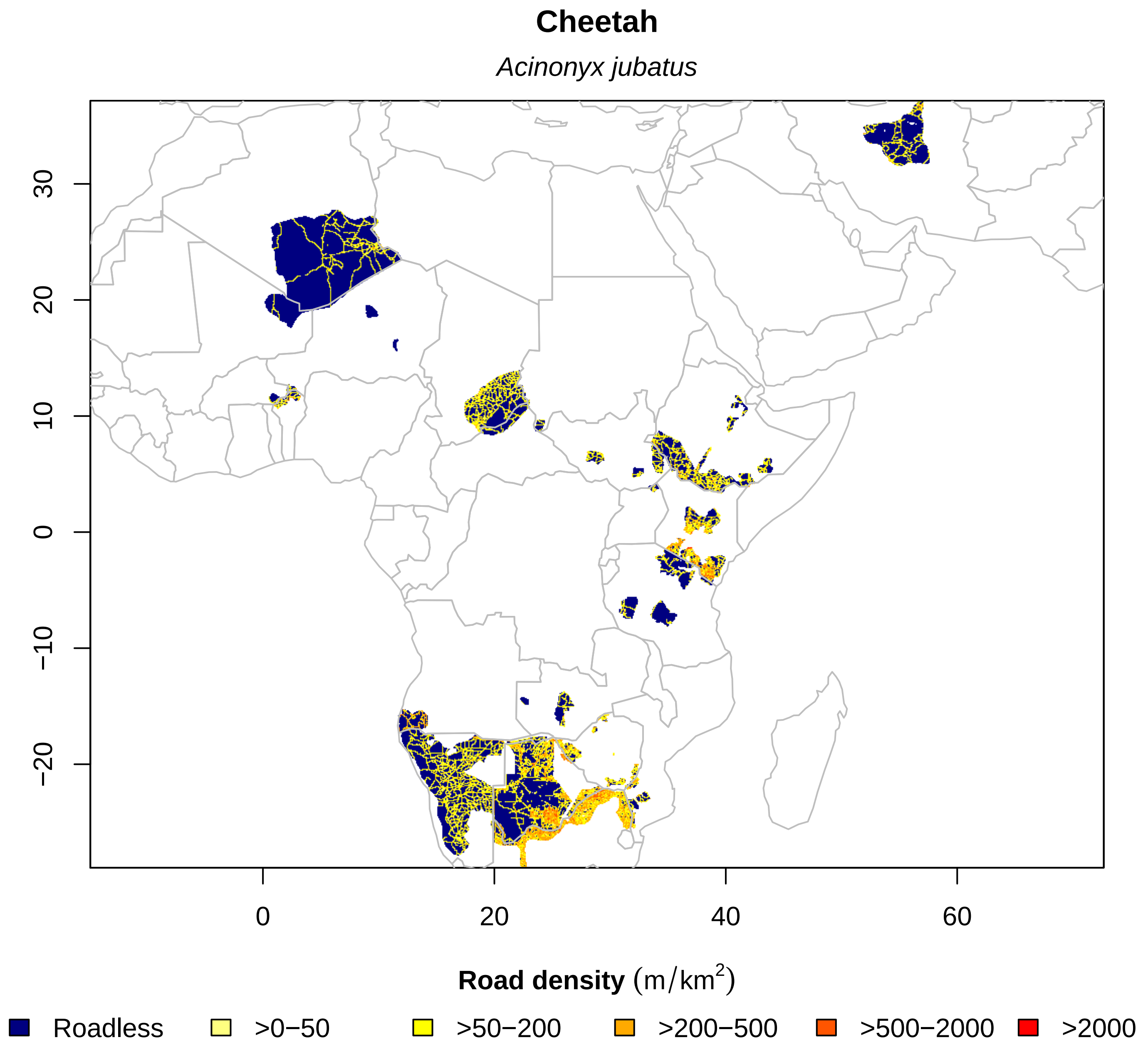
Cheetah
Acinonyx jubatus
30
20
10
0
−10
−20
0
20
40
60
Road density (m/km²)
Roadless
>0−50
>50−200
>200−500
>500−2000
>2000

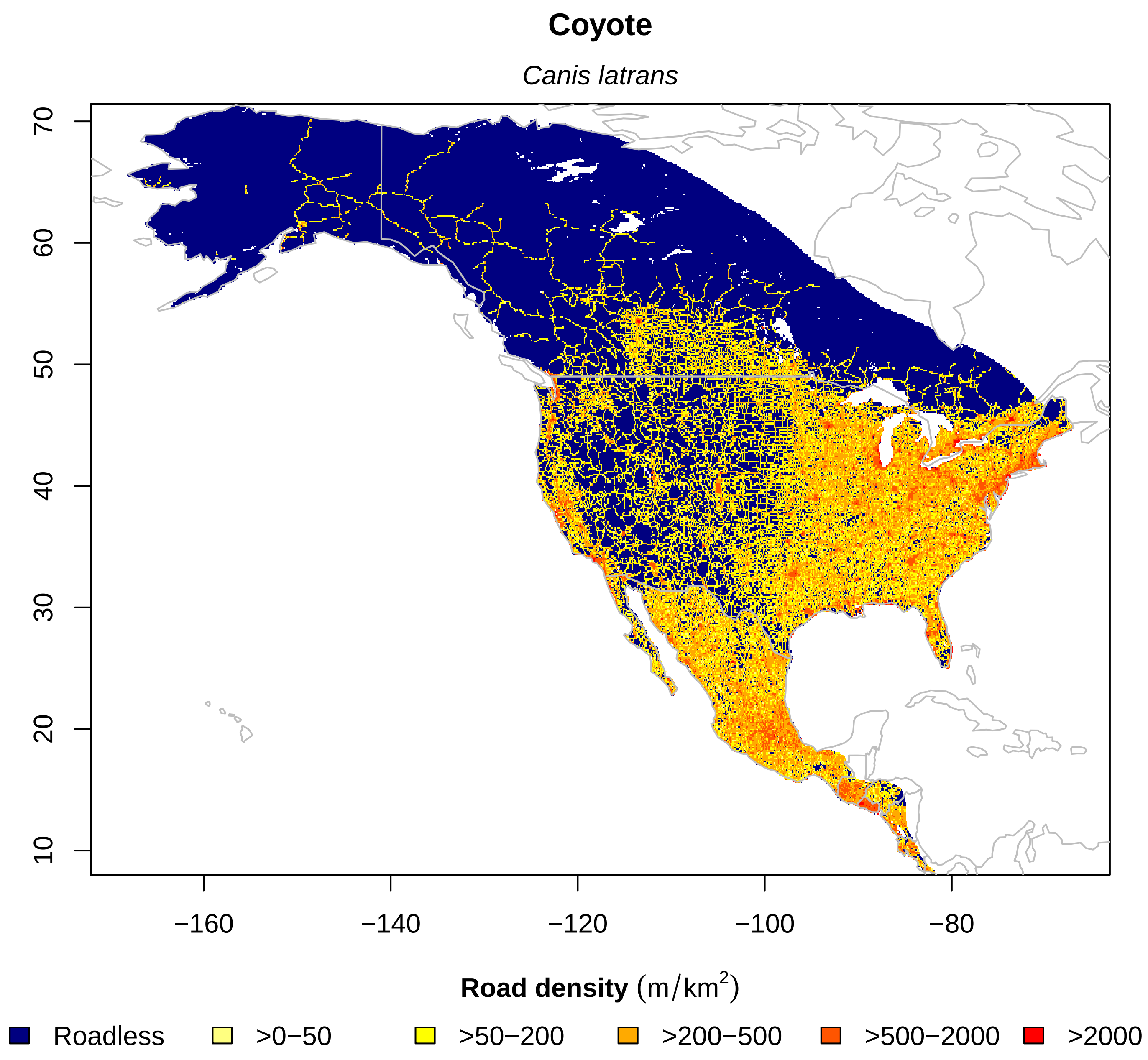

Coyote
Canis latrans
70
60
50
40
30
20
10
−160
−140
−120
−100
−80
Road density (m/km2)
Roadless
>0−50
>50−200
>200−500
>500−2000
>2000

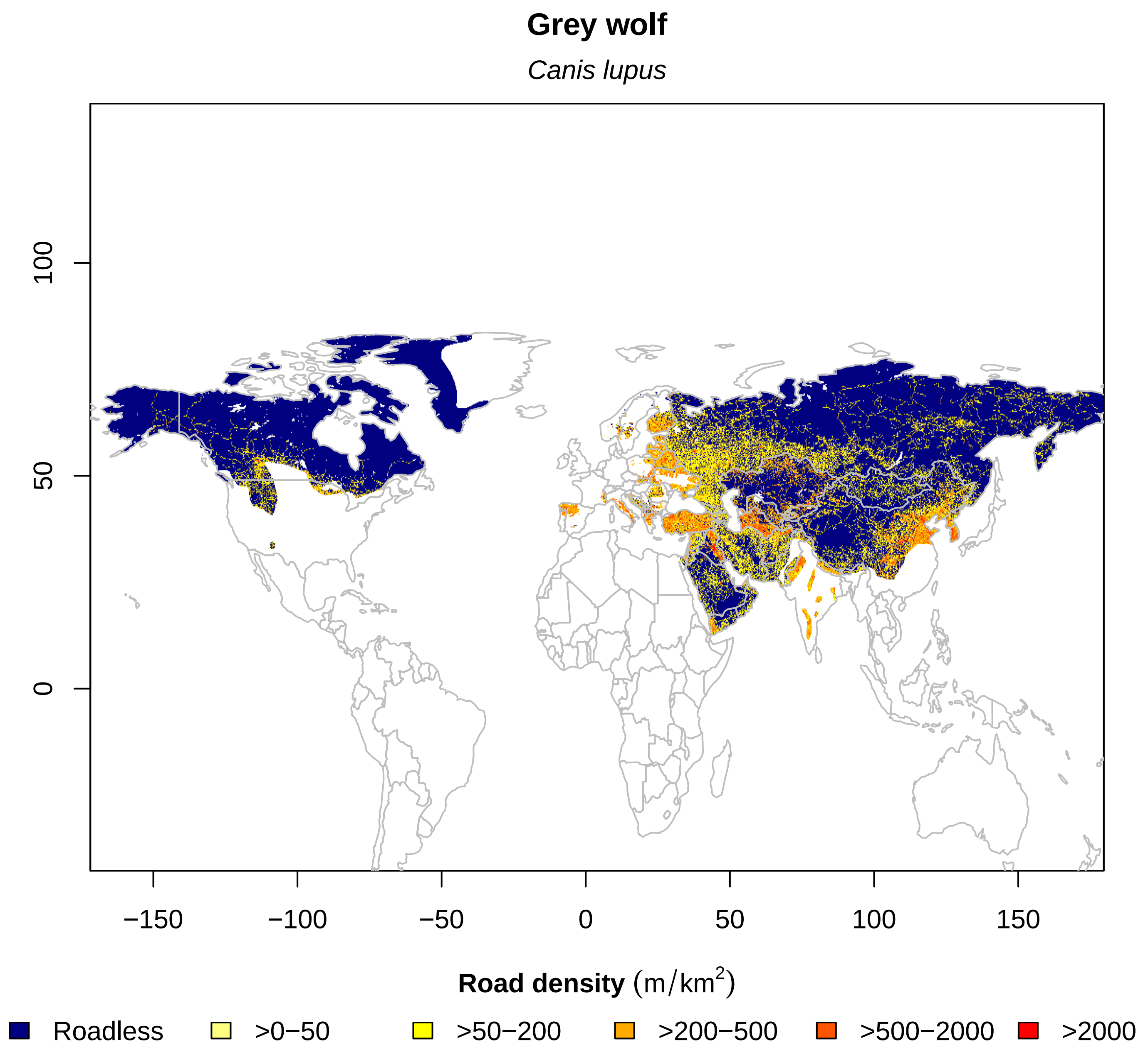

Grey wolf
Canis lupus
100
50
0
−150
−100
−50
0
50
100
150
Road density (m/km²)
Roadless
>0−50
>50−200
>200−500
>500−2000
>2000

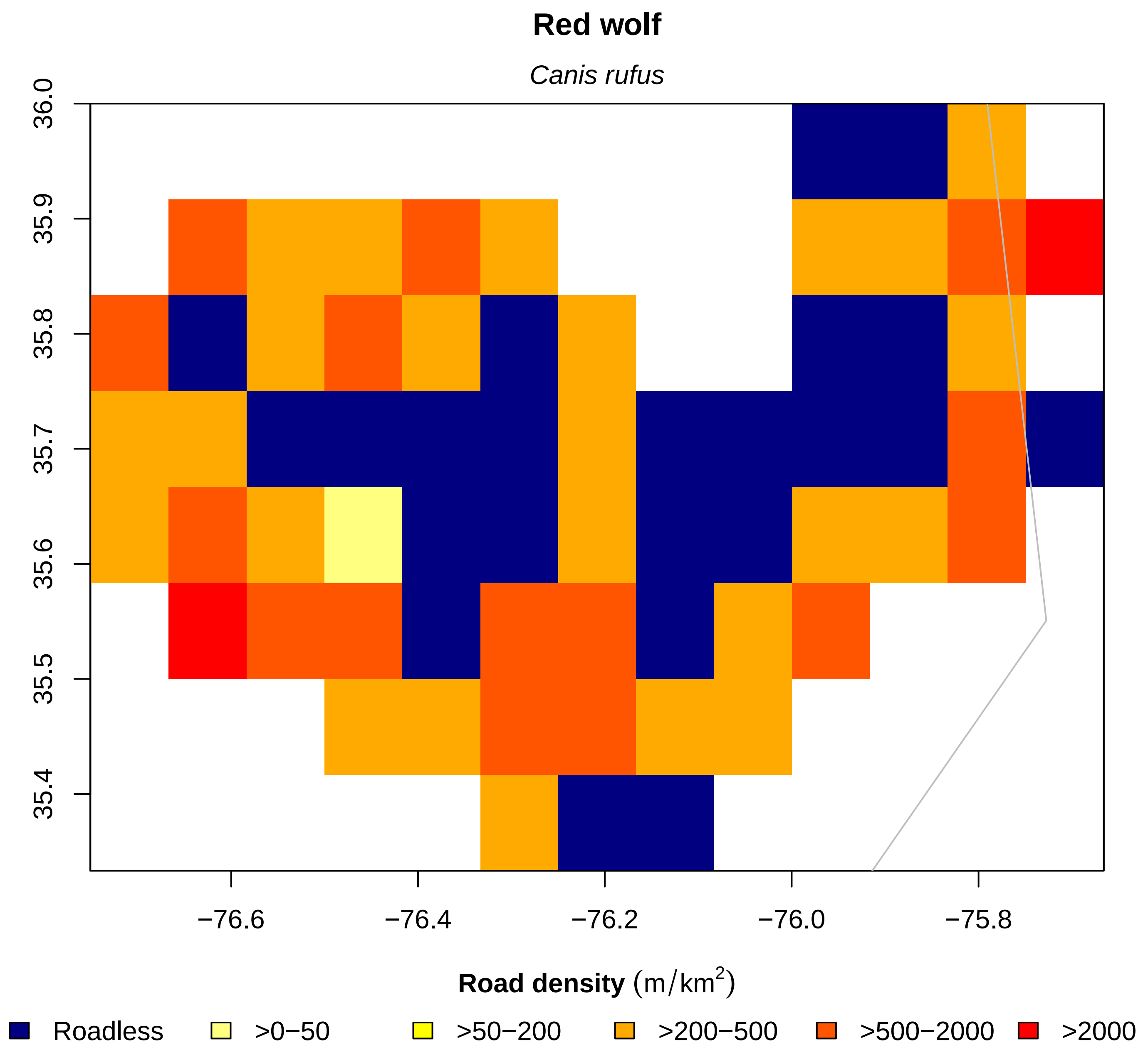

Red wolf
Canis rufus
36.0
35.9
35.8
35.7
35.6
35.5
35.4
−76.6
−76.4
−76.2
−76.0
−75.8
Road density (m/km²)
Roadless
>0−50
>50−200
>200−500
>500−2000
>2000

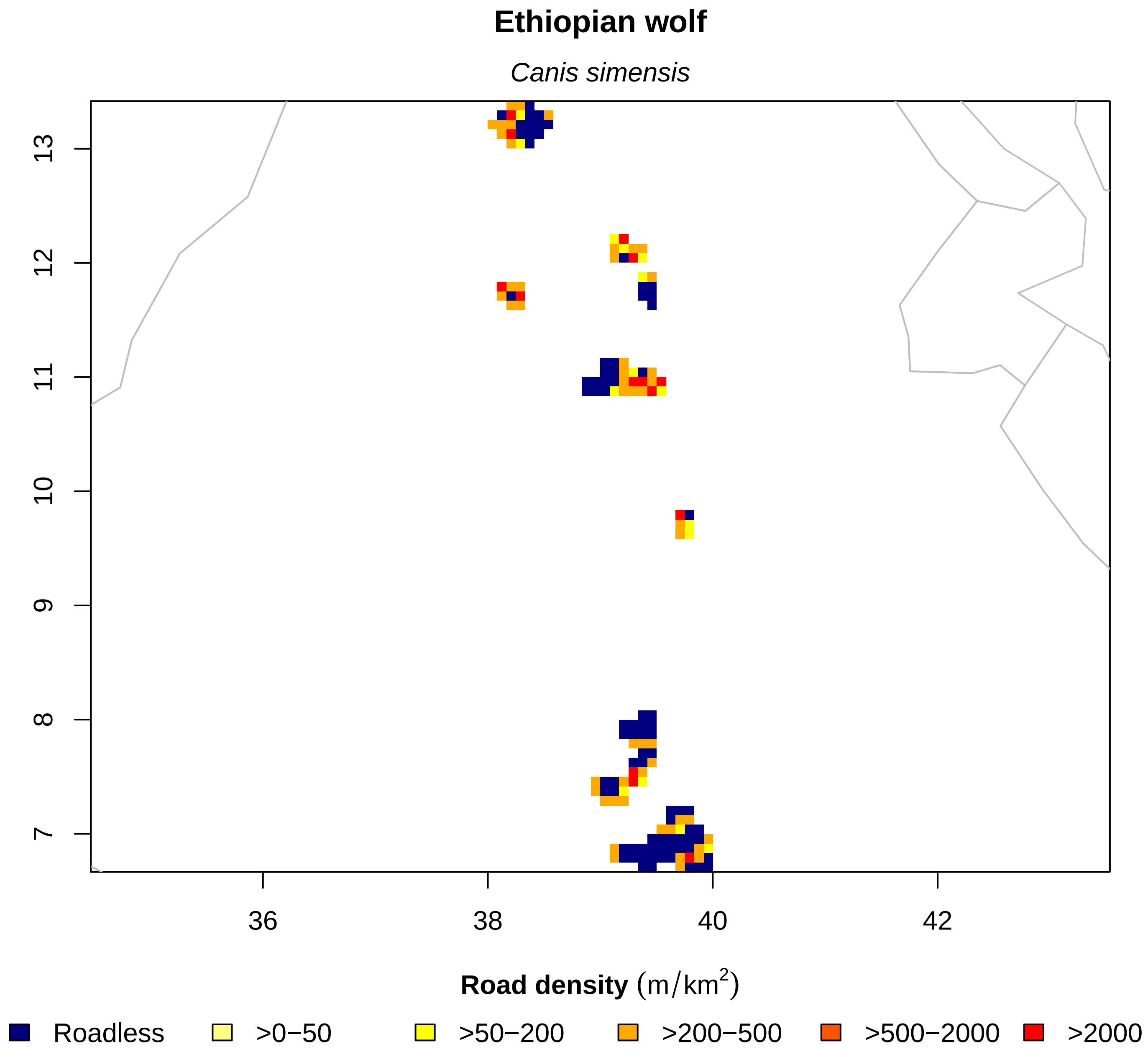

Ethiopian wolf
Canis simensis
13
12
11
10
9
8
7
36
38
40
42
Road density (m/km²)
Roadless
>0−50
>50−200
>200−500
>500−2000
>2000

Caracal

Caracal caracal

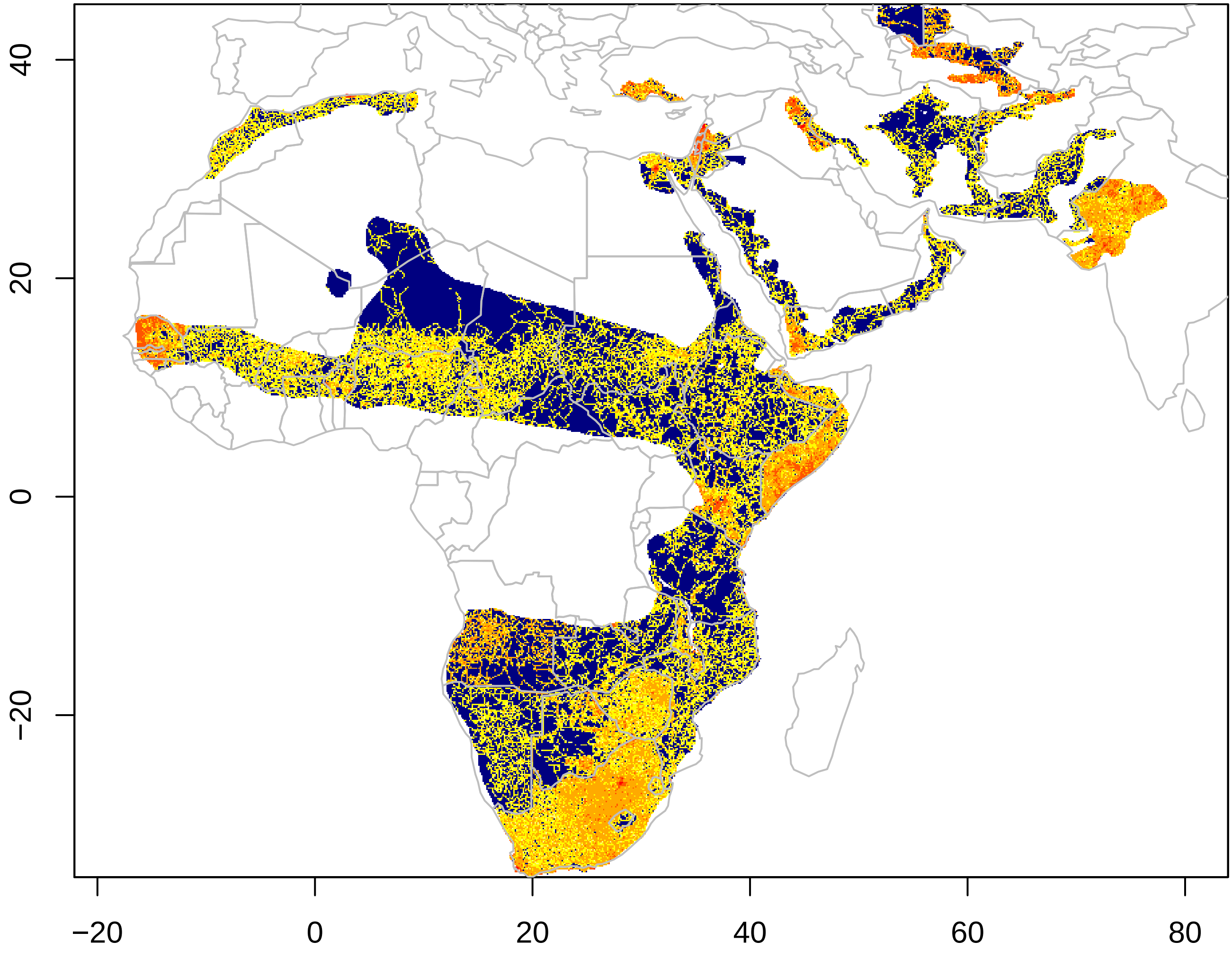

Road density (m/km^2)

Roadless >0−50 >50−200 >200−500 >500−2000 >2000

Asiatic golden cat

Catopuma temminckii

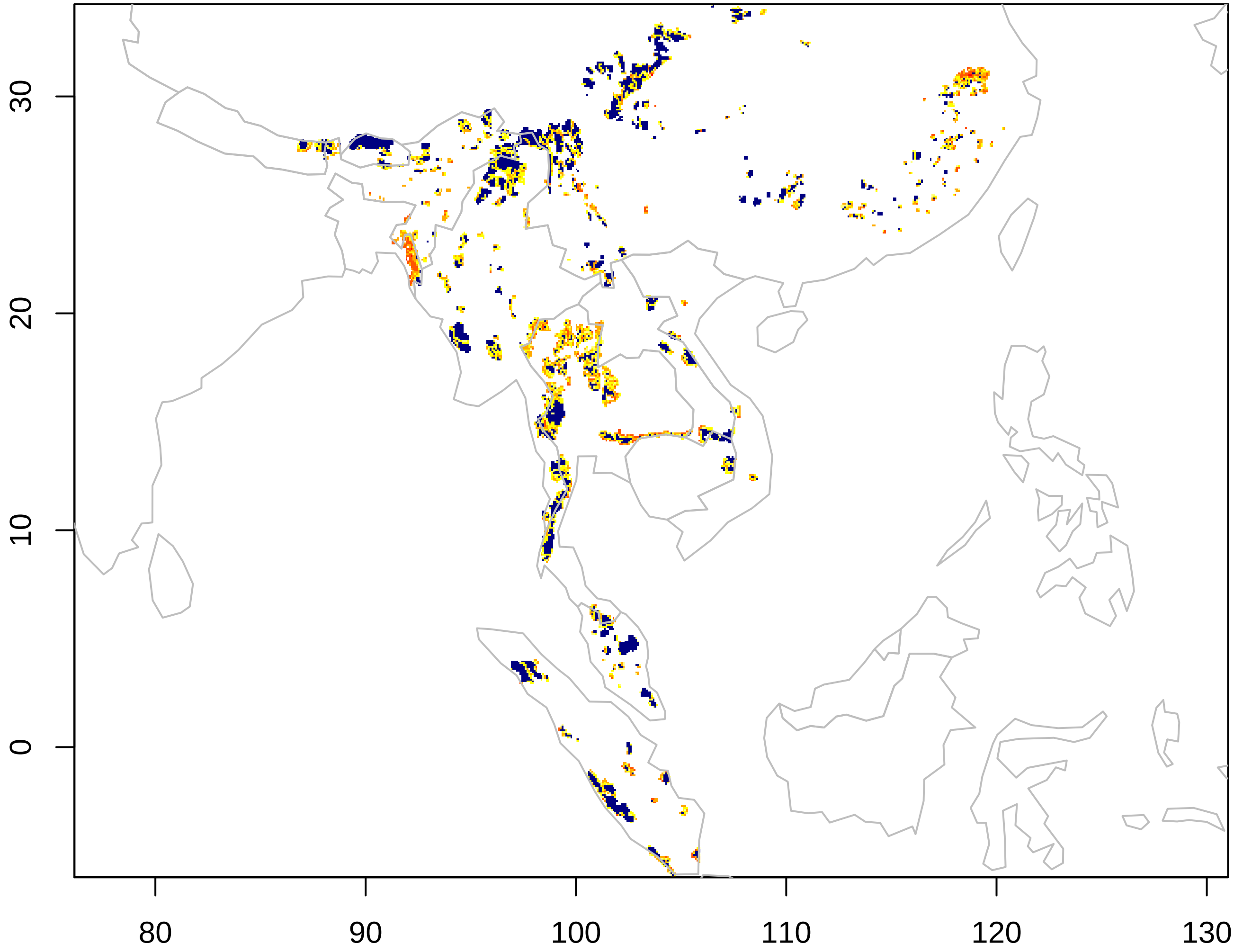

Road density (m/km^2)

Roadless >0−50 >50−200 >200−500 >500−2000 >2000

Maned wolf

Chrysocyon brachyurus

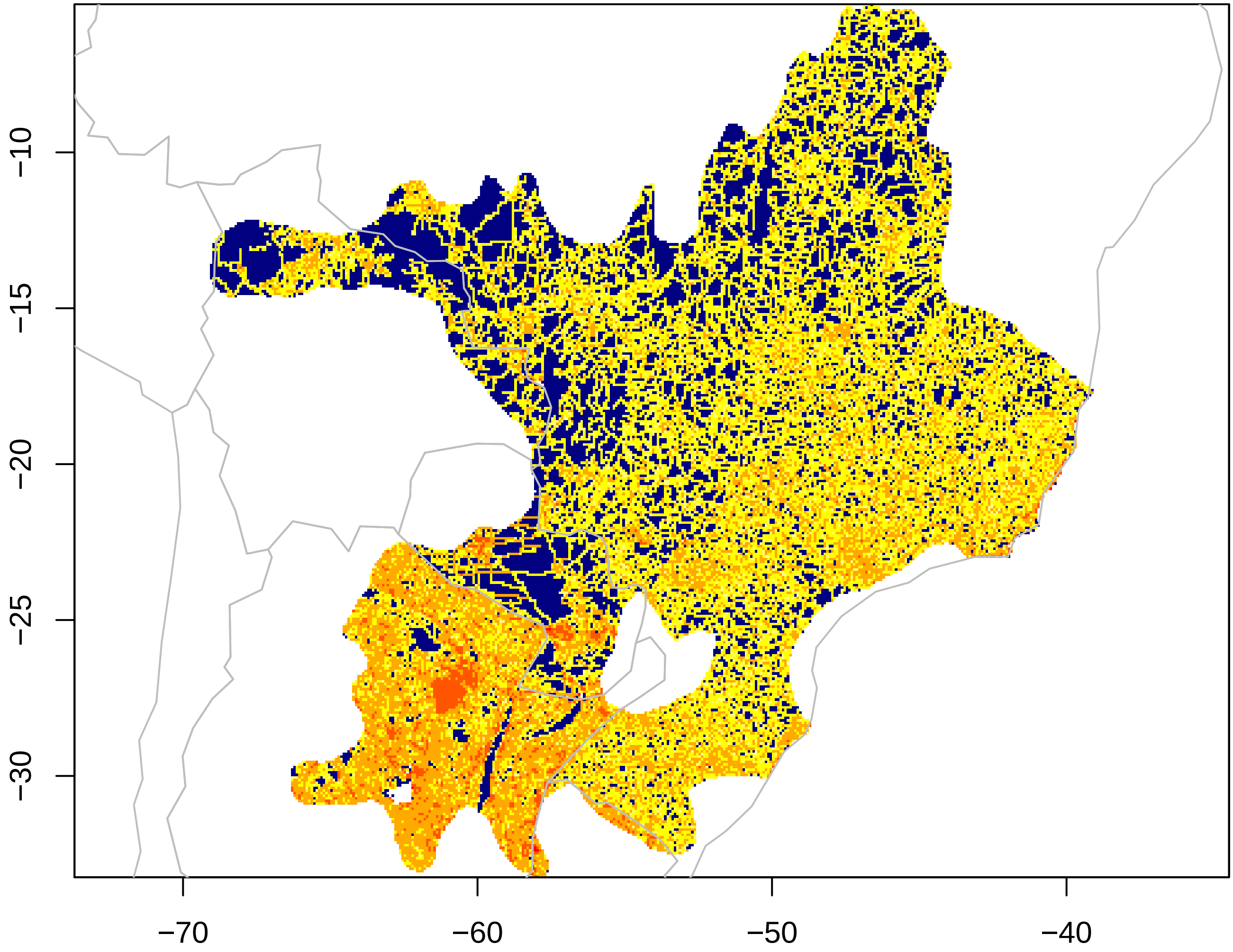

Road density (m/km^2)

Roadless | >0−50 | >50−200 | >200−500 | >500−2000 | >2000

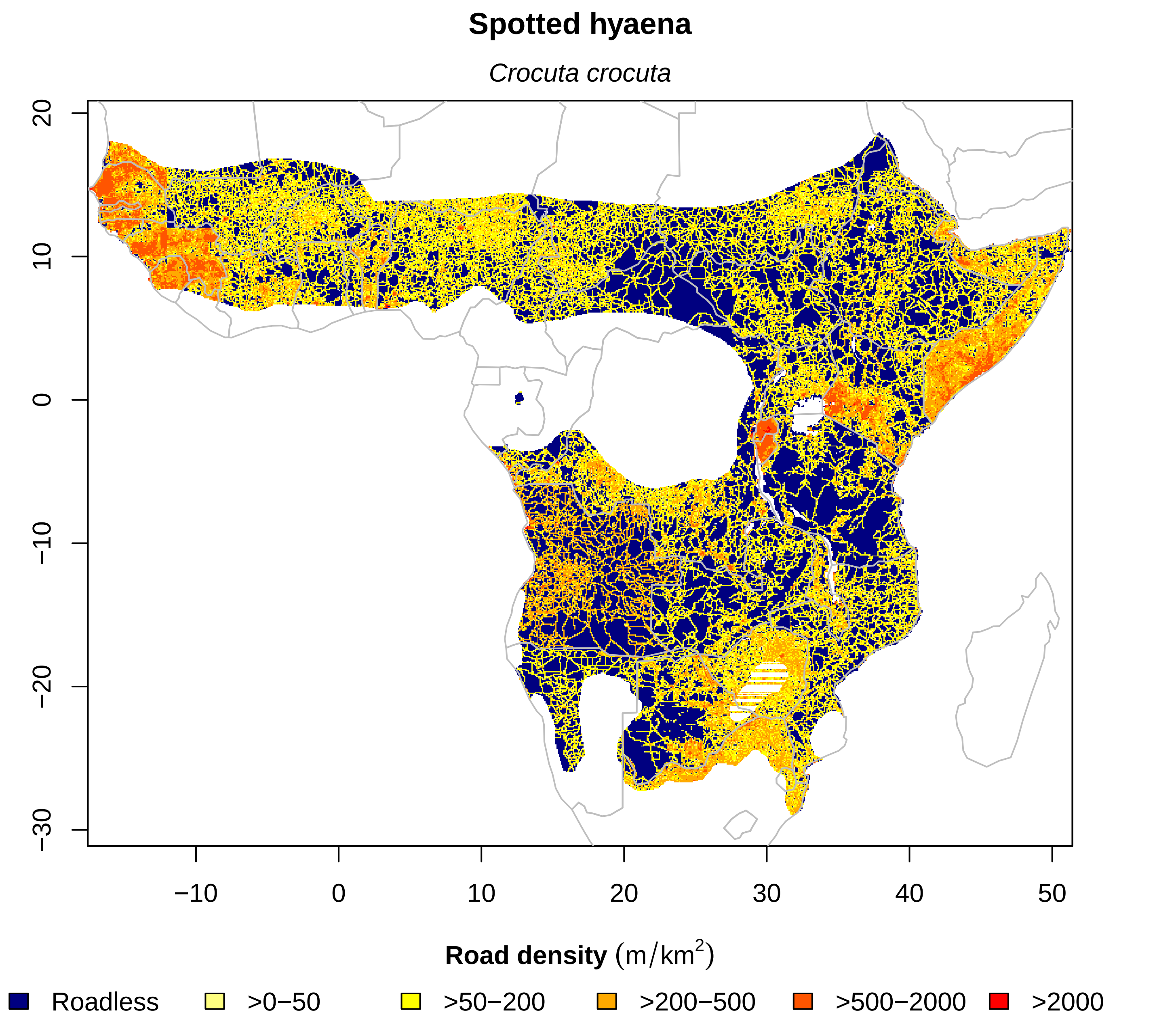
Spotted hyaena
Crocuta crocuta
20
10
0
−10
−20
−30
−10
0
10
20
30
40
50
Road density (m/km2)
Roadless
>0−50
>50−200
>200−500
>500−2000
>2000

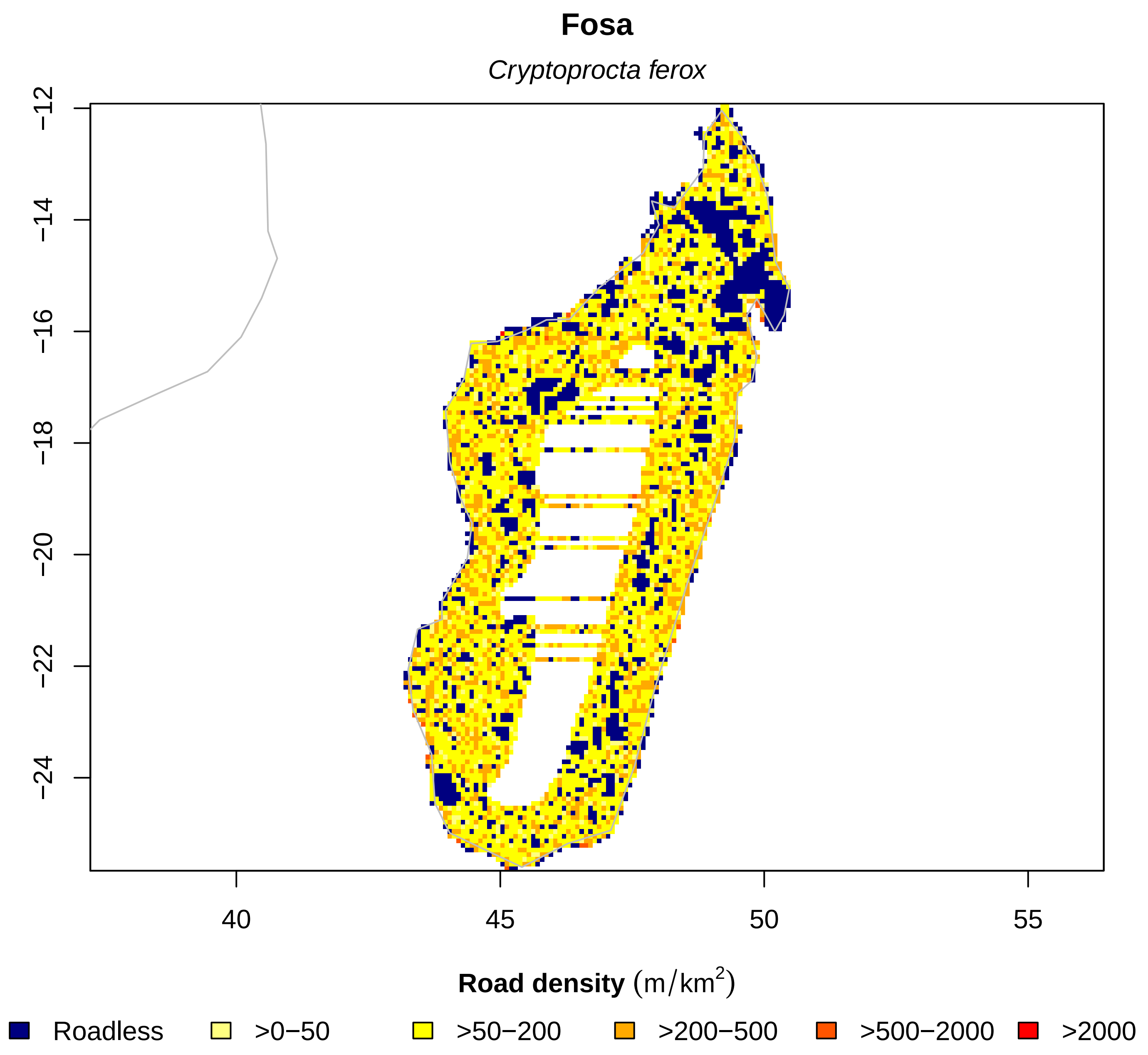
Fosa
Cryptoprocta ferox
−12
−14
−16
−18
−20
−22
−24
40
45
50
55
Road density (m/km2)
Roadless
>0−50
>50−200
>200−500
>500−2000
>2000

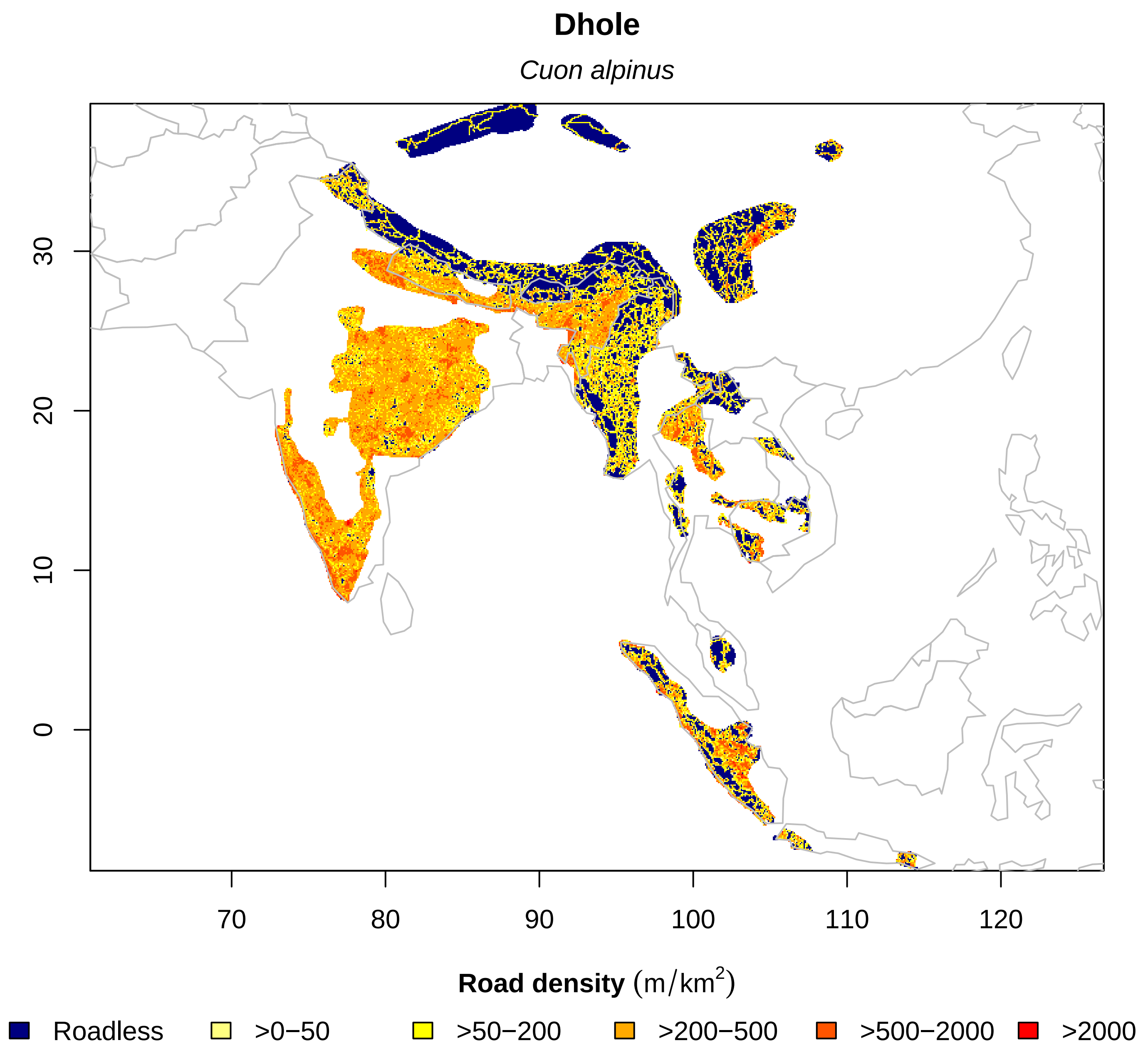
Dhole
Cuon alpinus
30
20
10
0
70
80
90
100
110
120
Road density (m/km2)
Roadless
>0−50
>50−200
>200−500
>500−2000
>2000

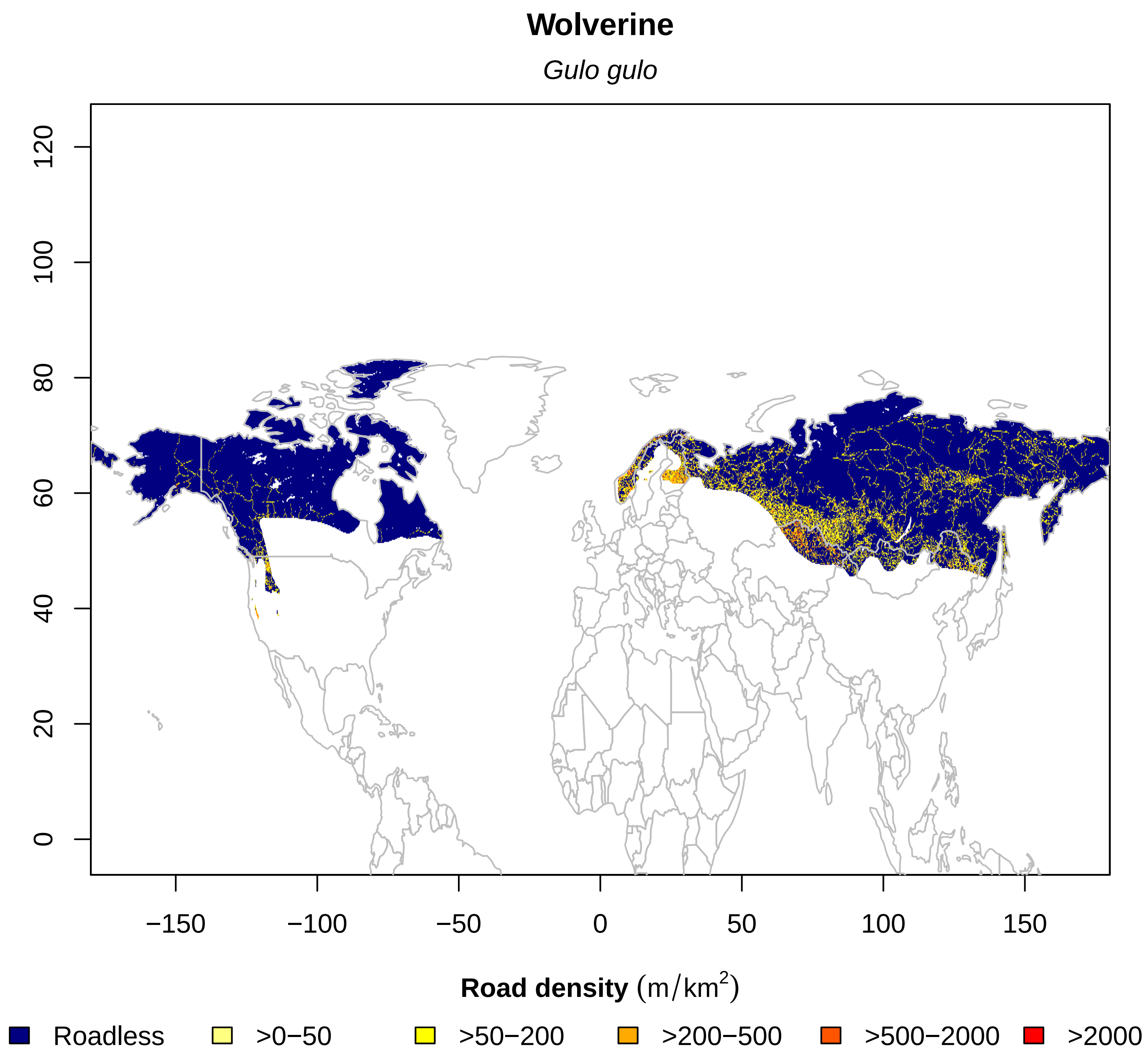
Wolverine
Gulo gulo
120
100
80
60
40
20
0
−150
−100
−50
0
50
100
150
Road density (m/km2)
Roadless
>0−50
>50−200
>200−500
>500−2000
>2000

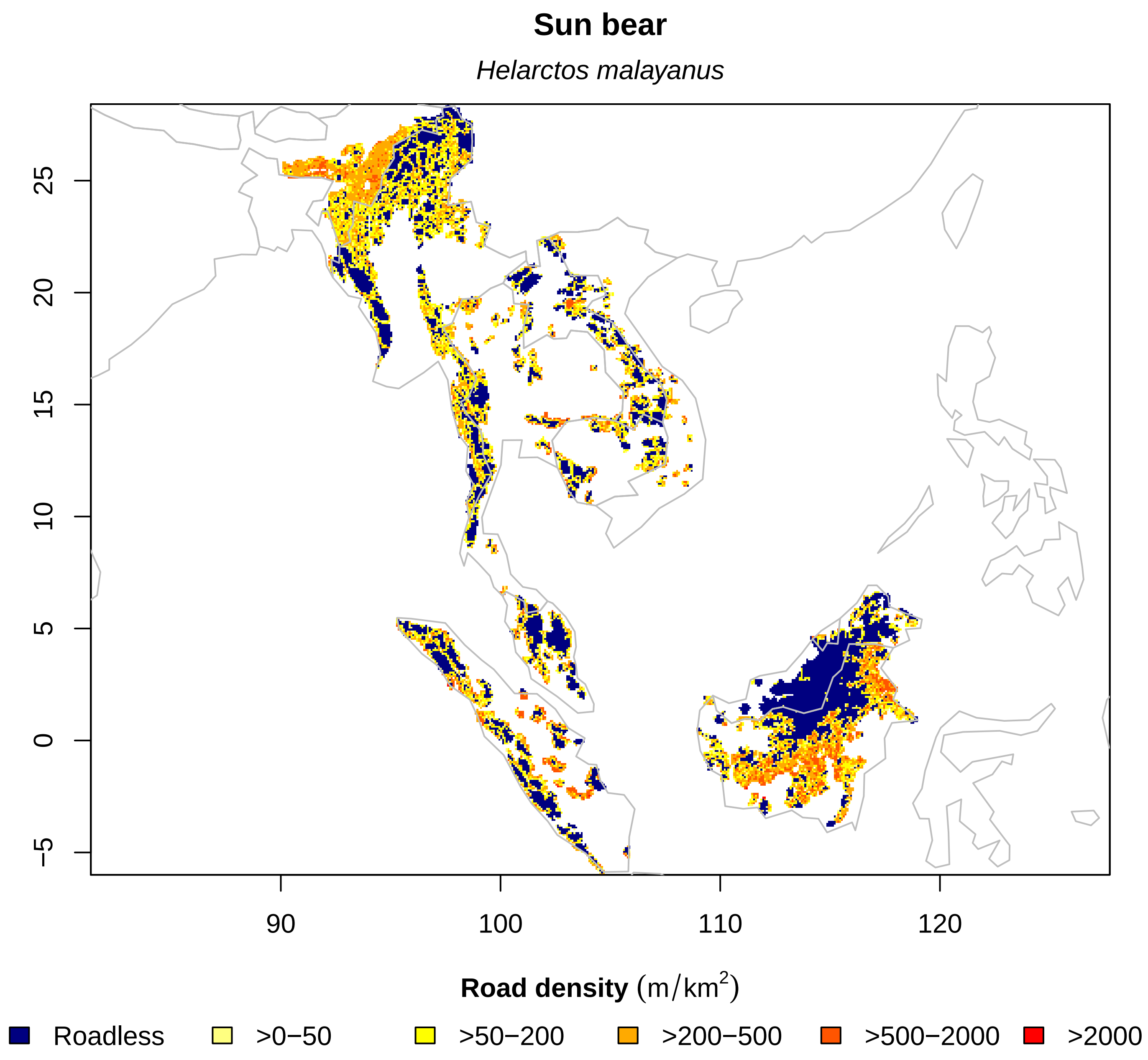

Sun bear
Helarctos malayanus
25
20
15
10
5
0
−5
90
100
110
120
Road density (m/km2)
Roadless
>0−50
>50−200
>200−500
>500−2000
>2000

Striped hyaena

Hyaena hyaena

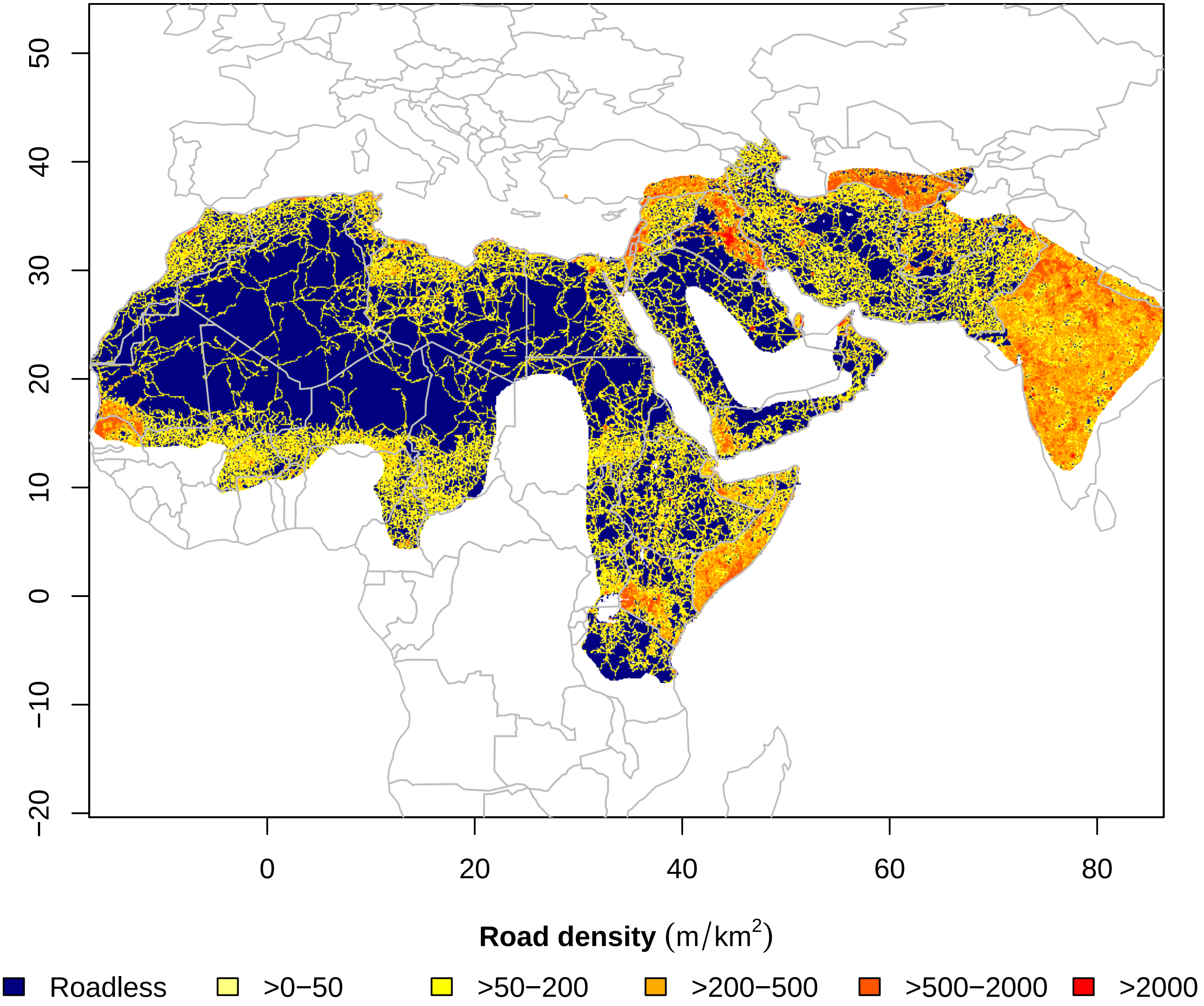

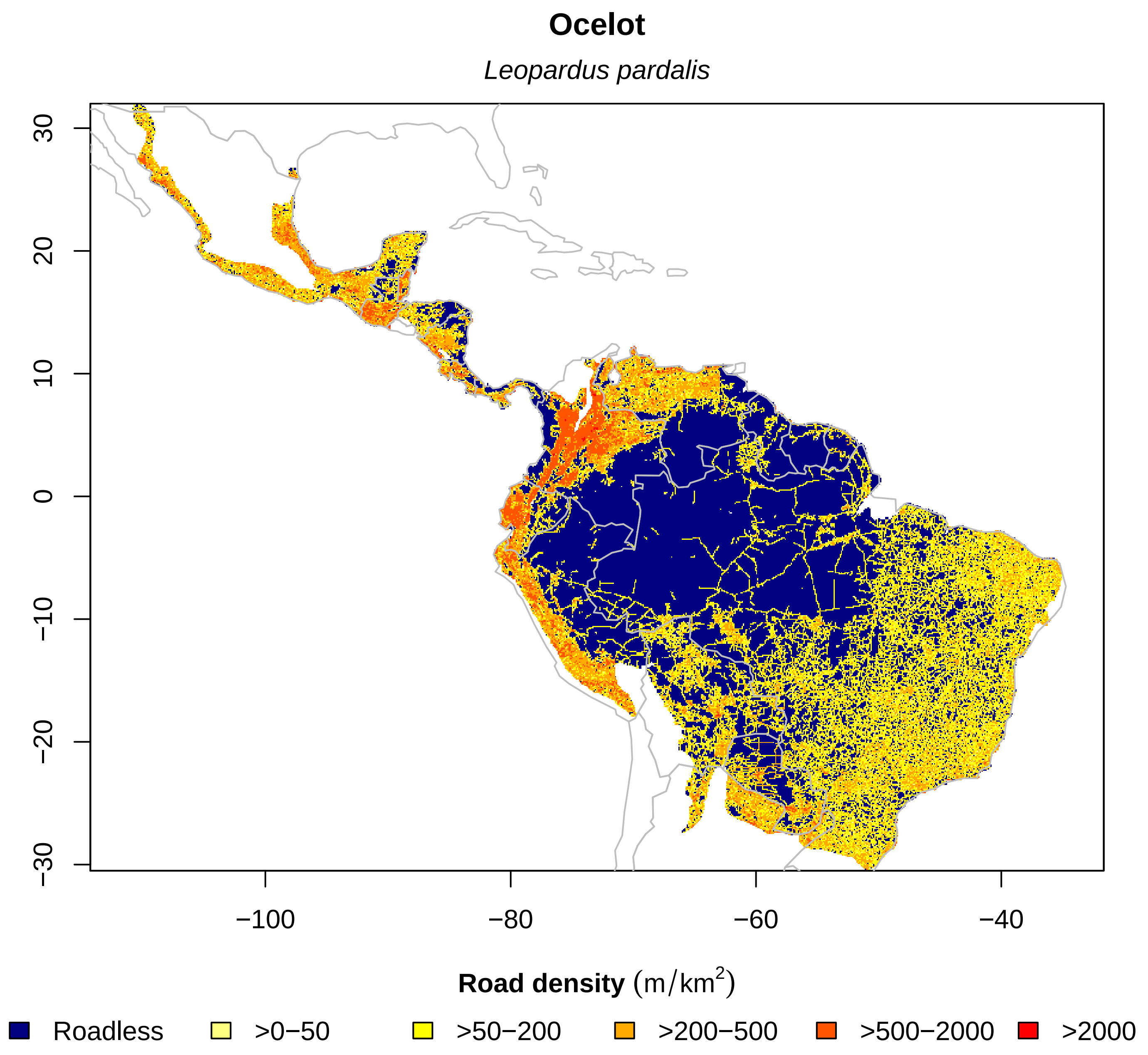

Ocelot
Leopardus pardalis
30
20
10
0
−10
−20
−30
−100
−80
−60
−40
Road density (m/km^2)
Roadless
>0−50
>50−200
>200−500
>500−2000
>2000

Serval

Leptailurus serval

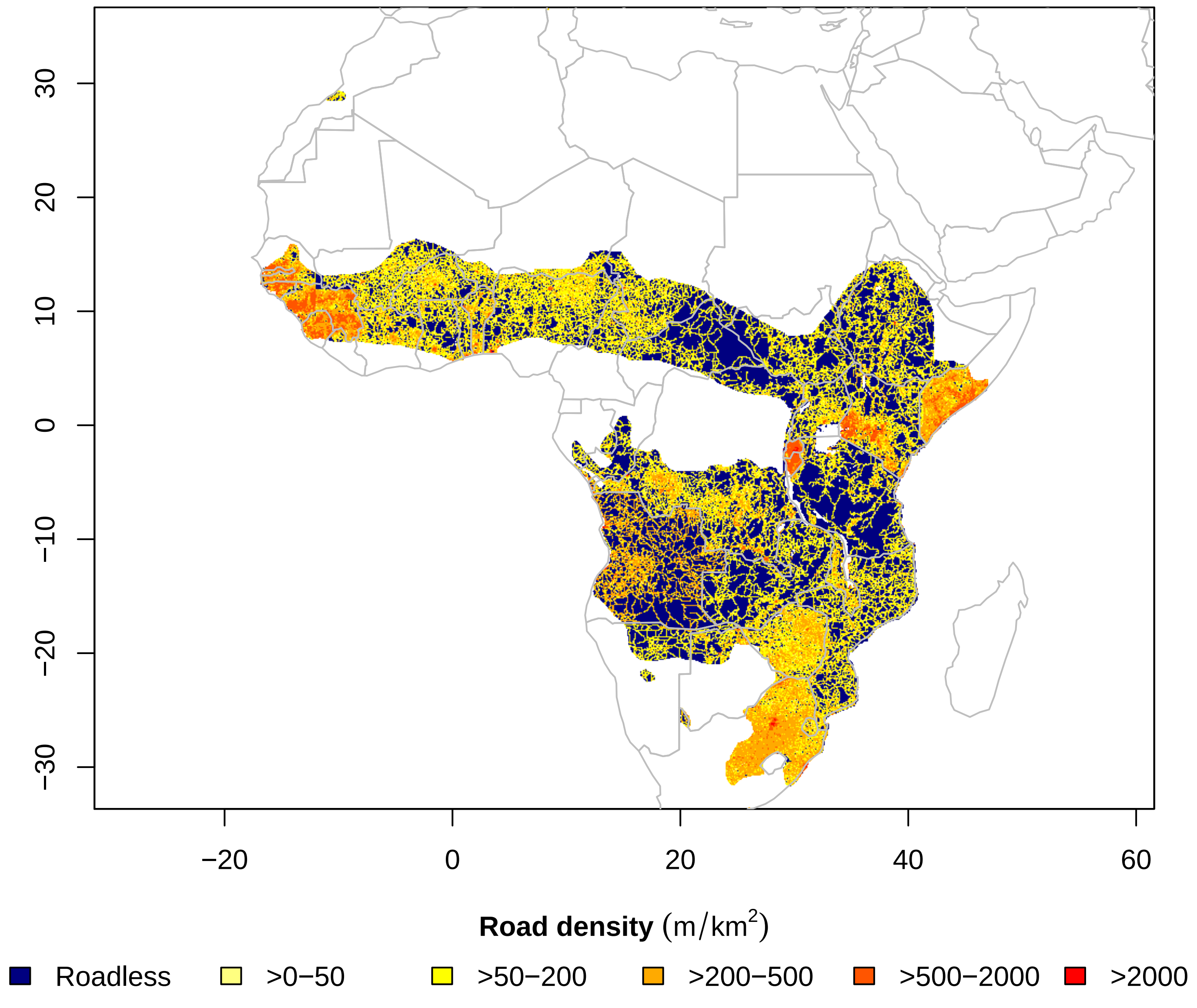

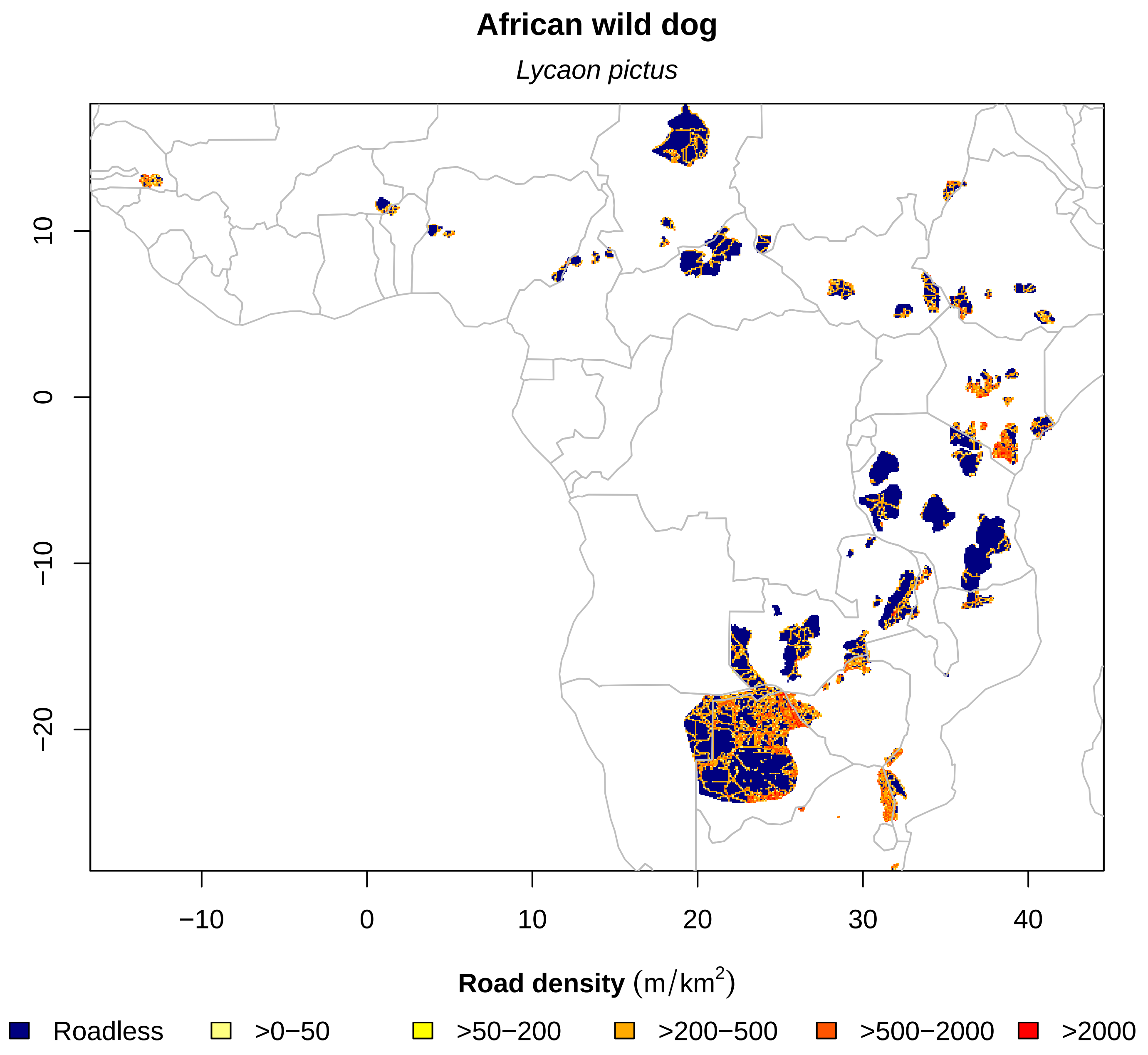

African wild dog
Lycaon pictus
10
0
−10
−20
−10
0
10
20
30
40
Road density (m/km2)
Roadless
>0−50
>50−200
>200−500
>500−2000
>2000

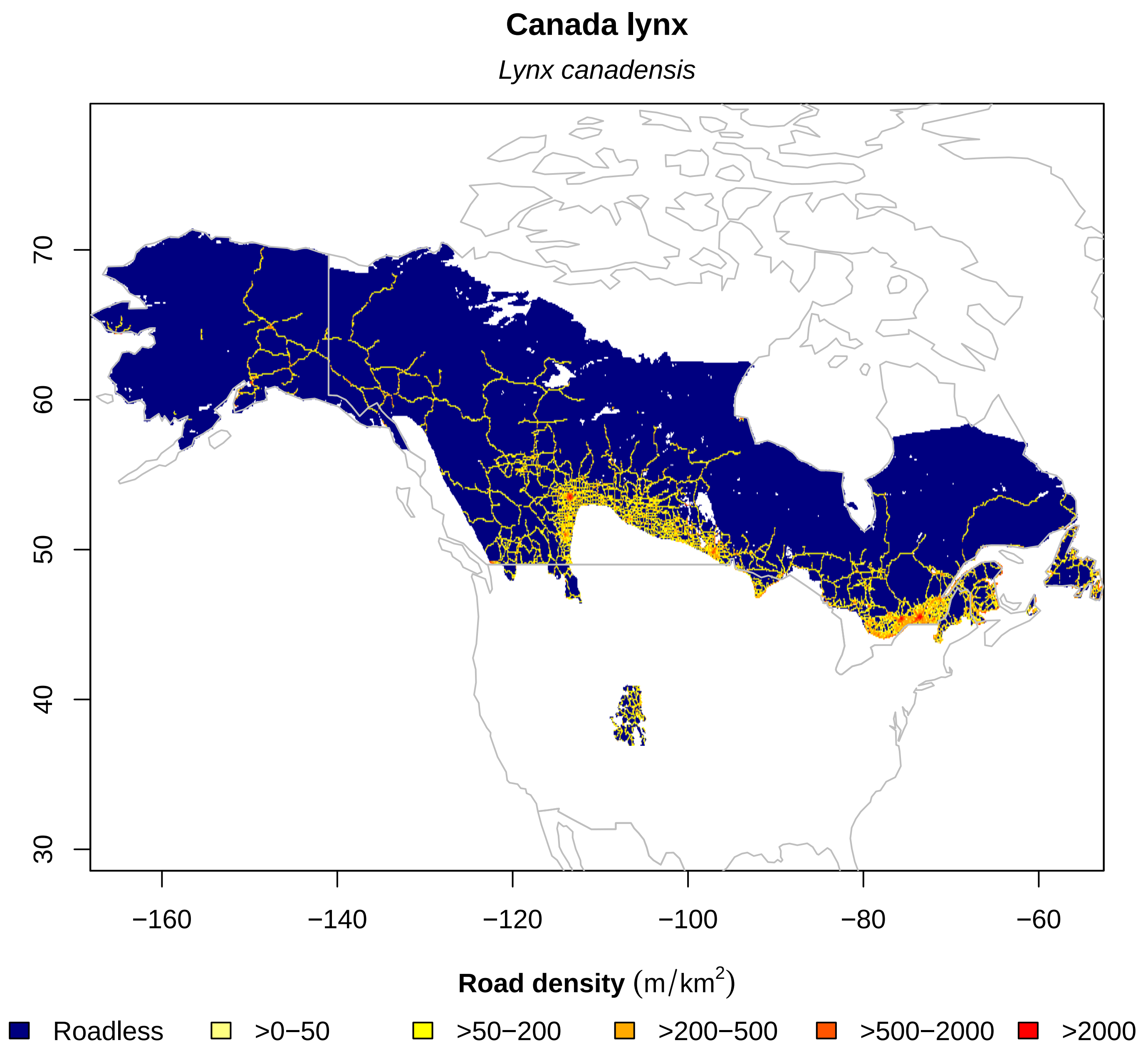

Canada lynx
Lynx canadensis
70
60
50
40
30
−160
−140
−120
−100
−80
−60
Road density (m/km2)
Roadless
>0−50
>50−200
>200−500
>500−2000
>2000

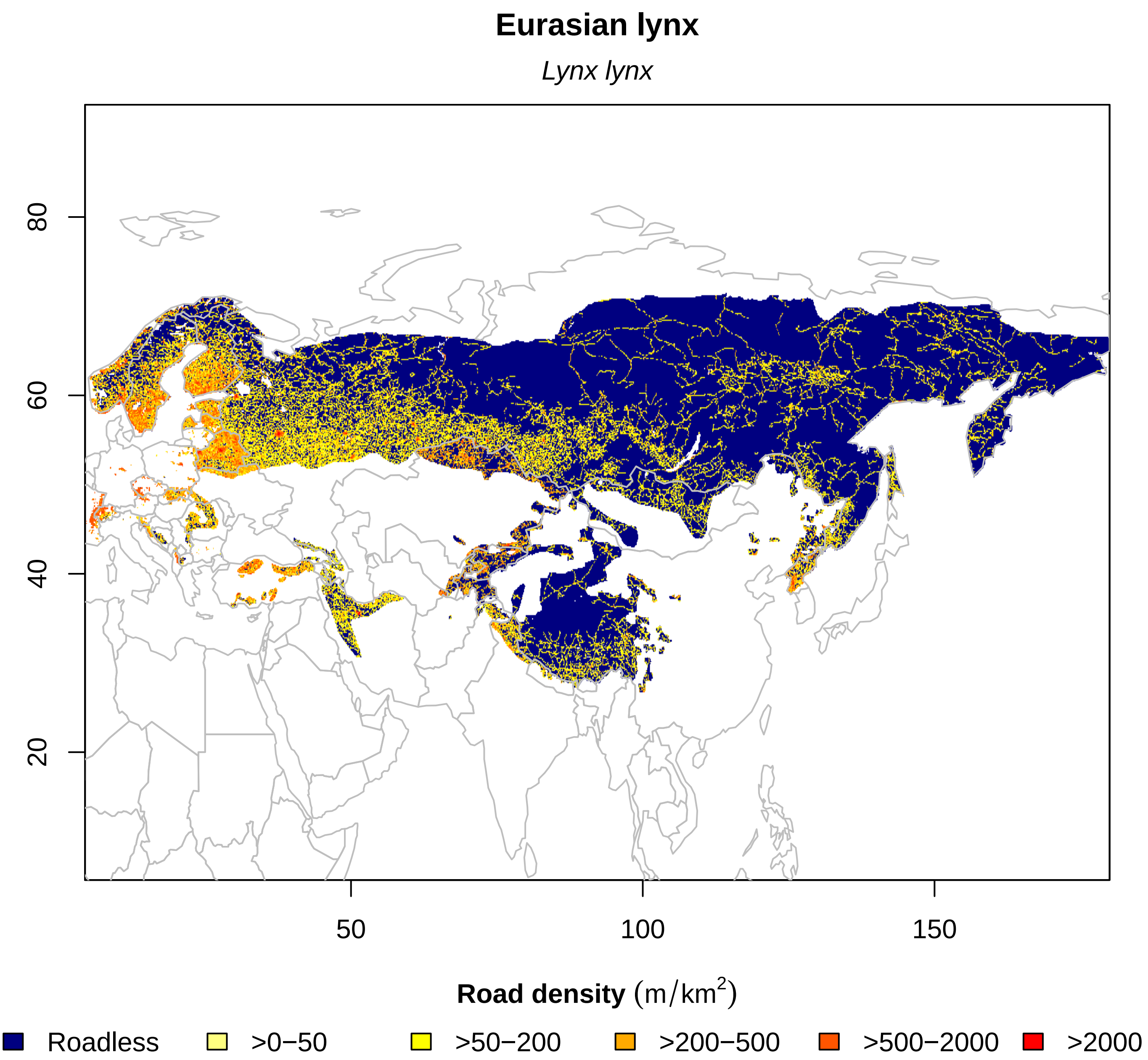
Eurasian lynx
Lynx lynx
80
60
40
20
50
100
150
Road density (m/km2)
Roadless
>0−50
>50−200
>200−500
>500−2000
>2000

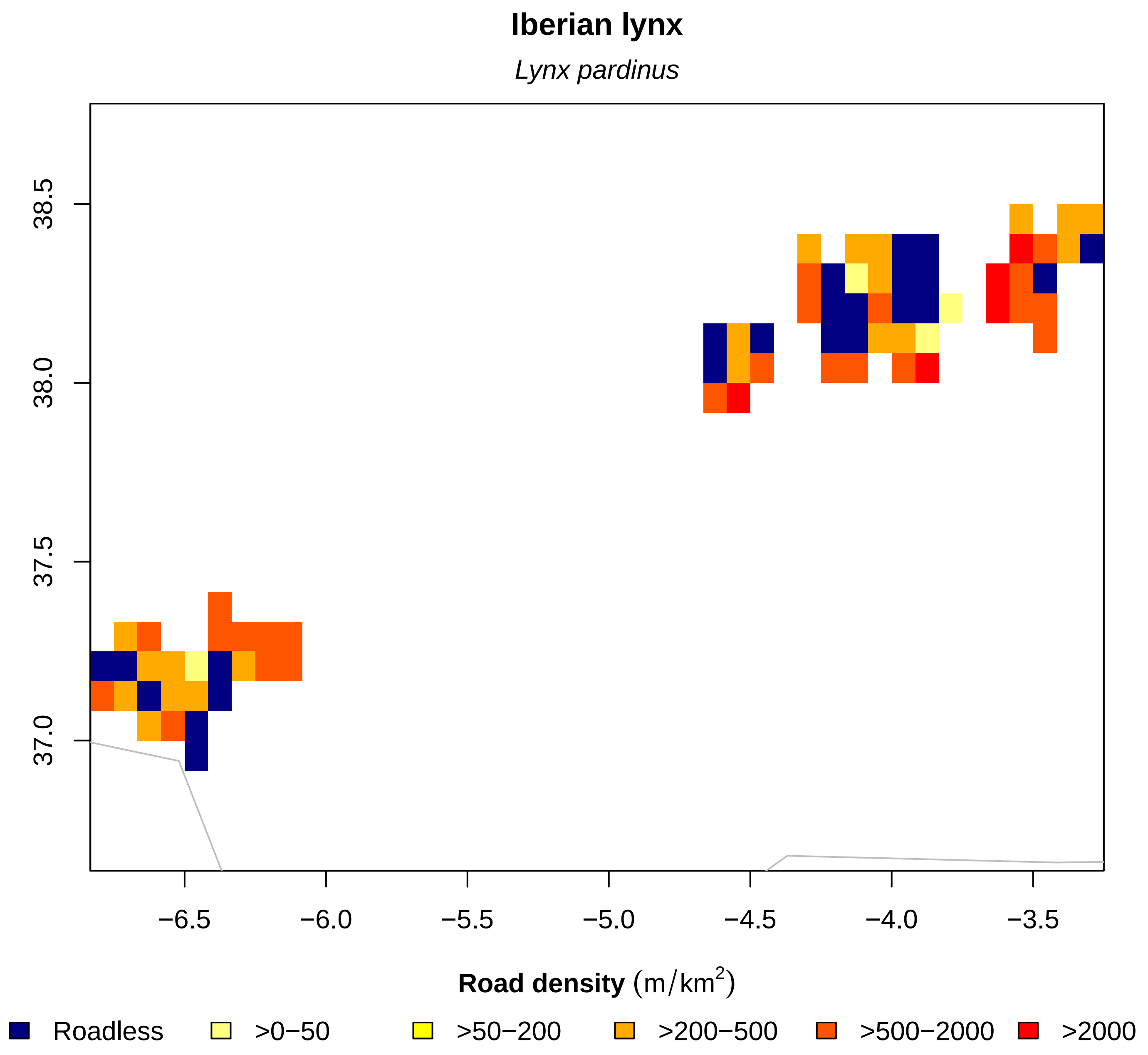
Iberian lynx
Lynx pardinus
38.5
38.0
37.5
37.0
−6.5
−6.0
−5.5
−5.0
−4.5
−4.0
−3.5
Road density (m/km²)
Roadless
>0−50
>50−200
>200−500
>500−2000
>2000

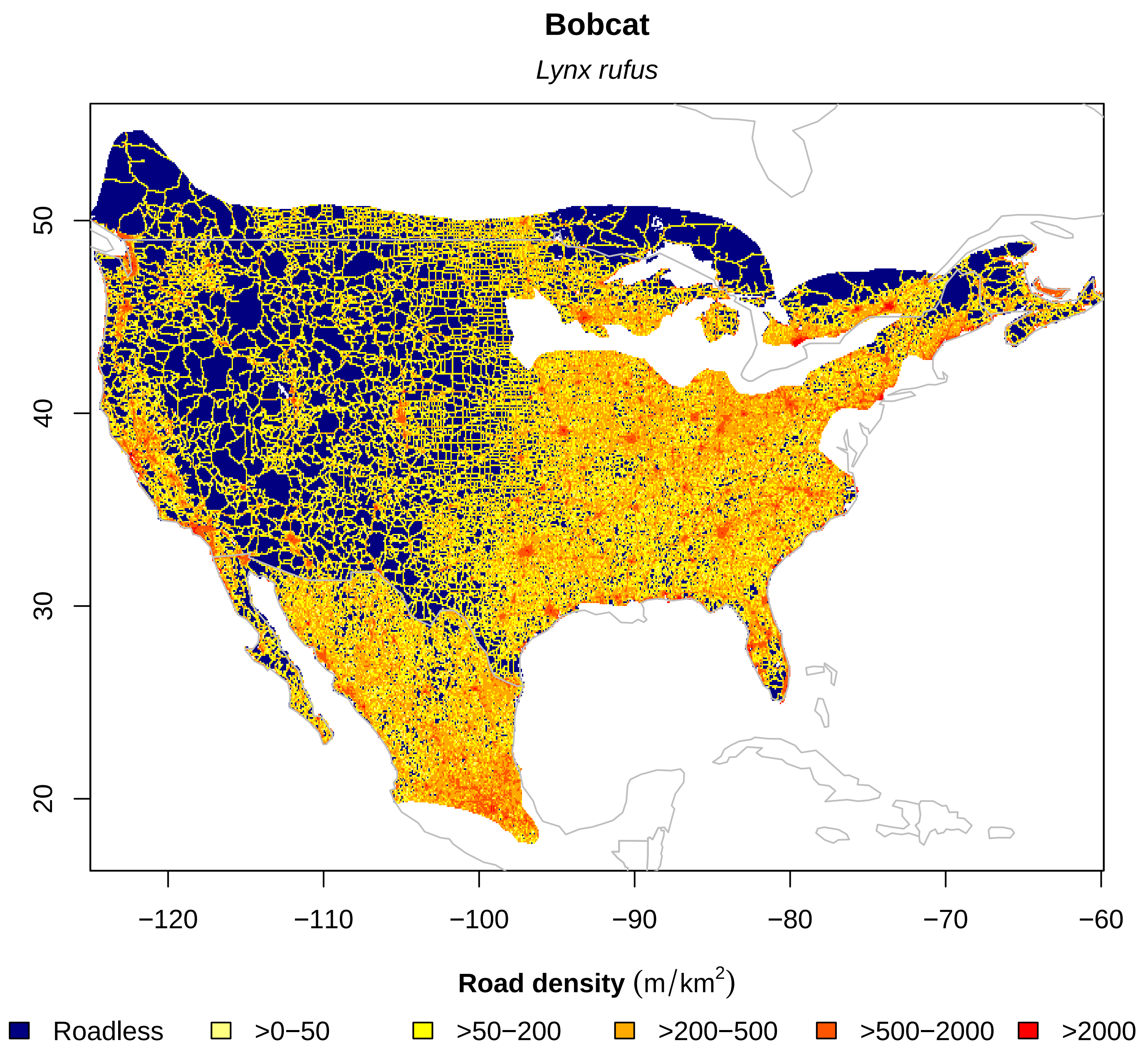

Bobcat
Lynx rufus
50
40
30
20
−120
−110
−100
−90
−80
−70
−60
Road density (m/km2)
Roadless
>0−50
>50−200
>200−500
>500−2000
>2000

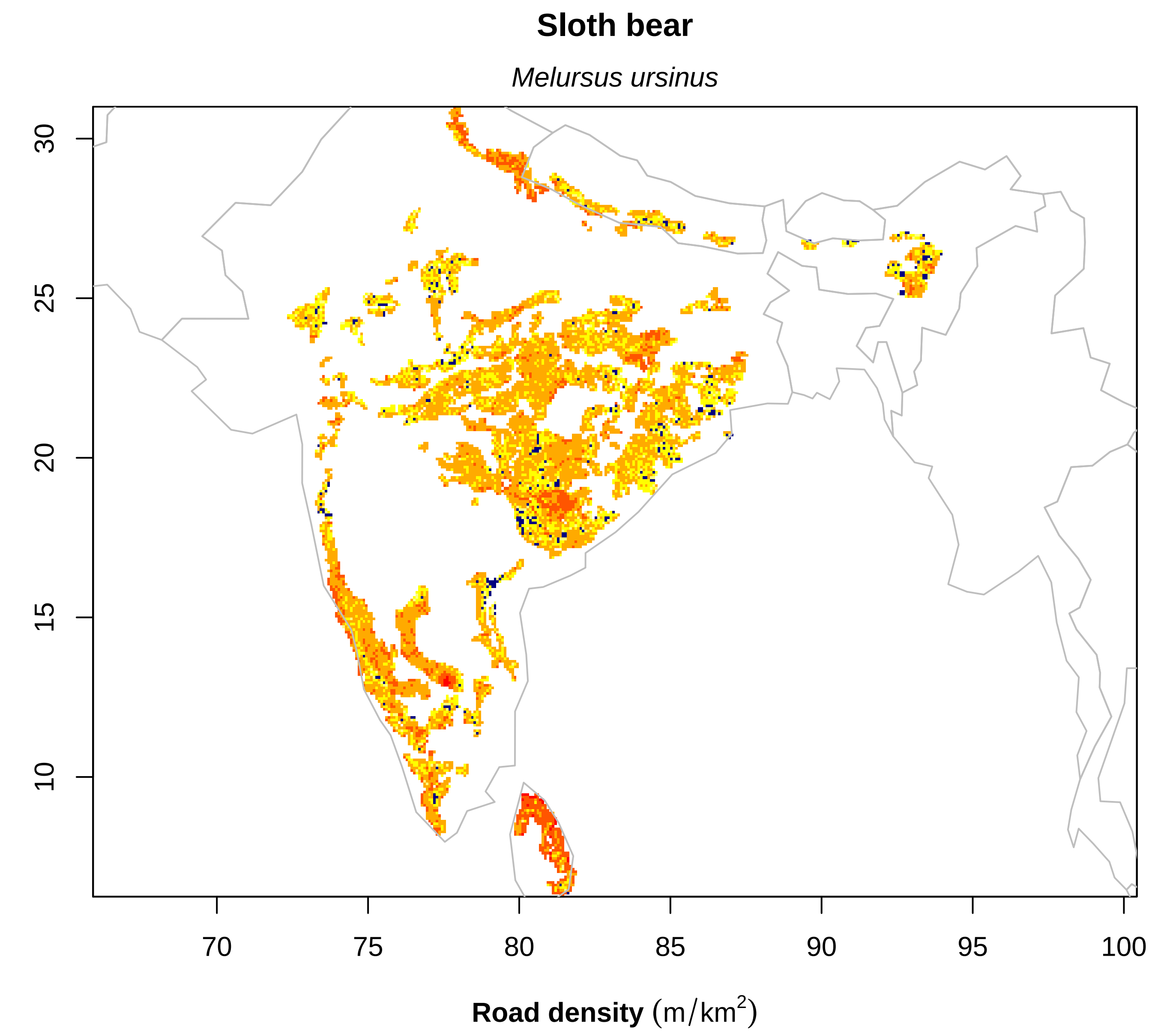

Sloth bear
Melursus ursinus
30
25
20
15
10
70
75
80
85
90
95
100
Road density (m/km^2)
Roadless
>0−50
>50−200
>200−500
>500−2000
>2000

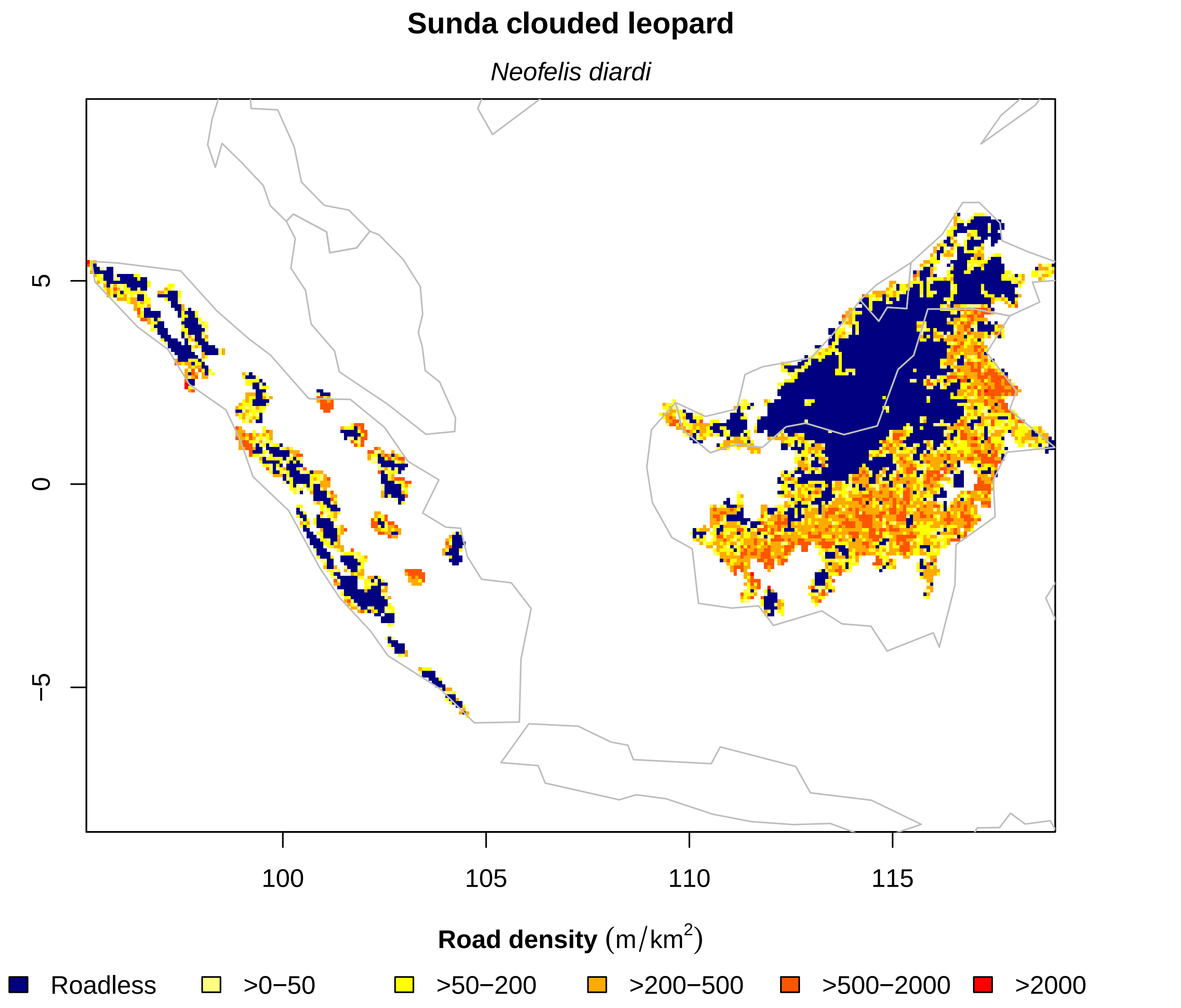

Sunda clouded leopard
Neofelis diardi
5
0
−5
100
105
110
115
Road density (m/km2)
Roadless
>0−50
>50−200
>200−500
>500−2000
>2000

Clouded loepard

Neofelis nebulosa

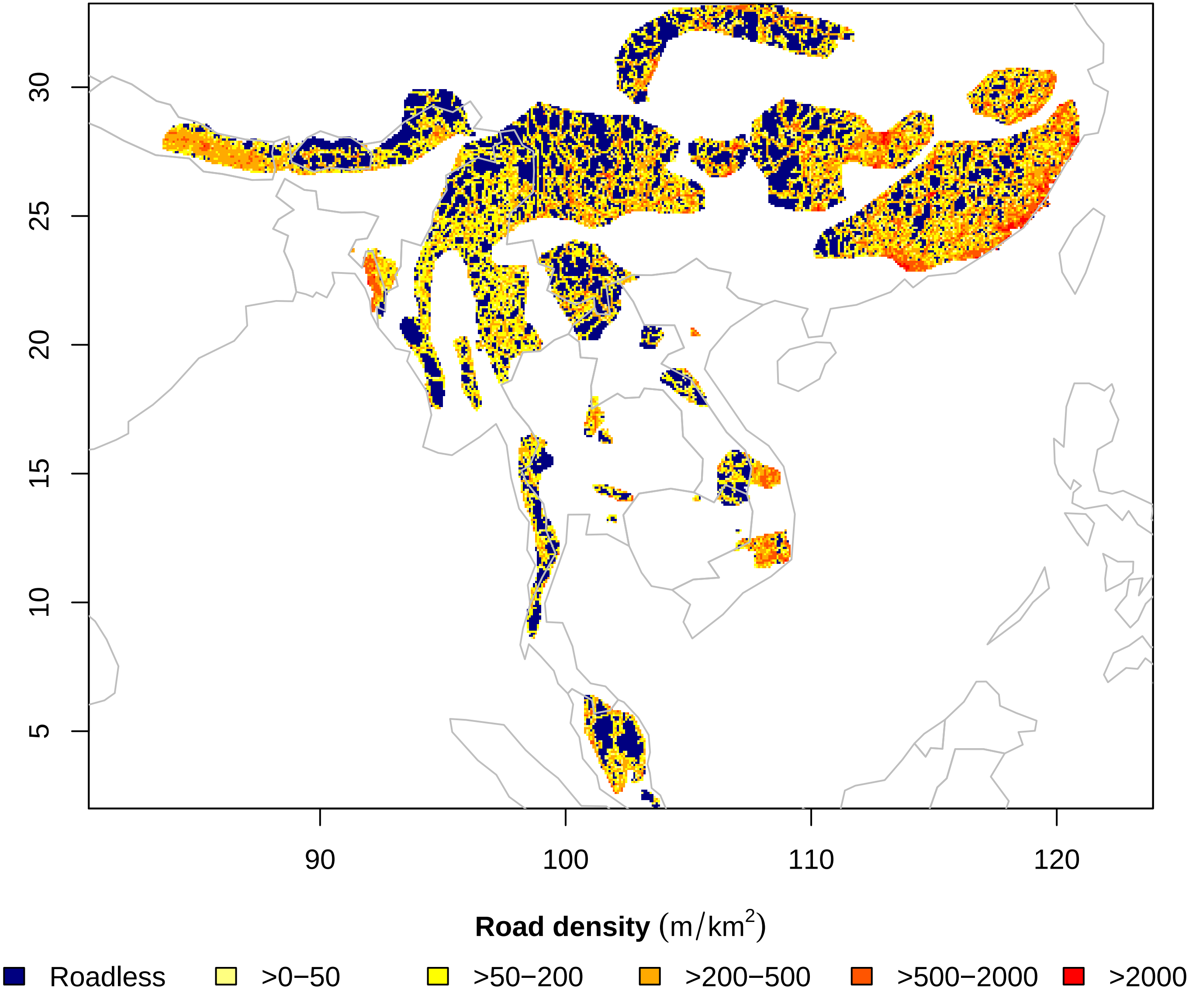

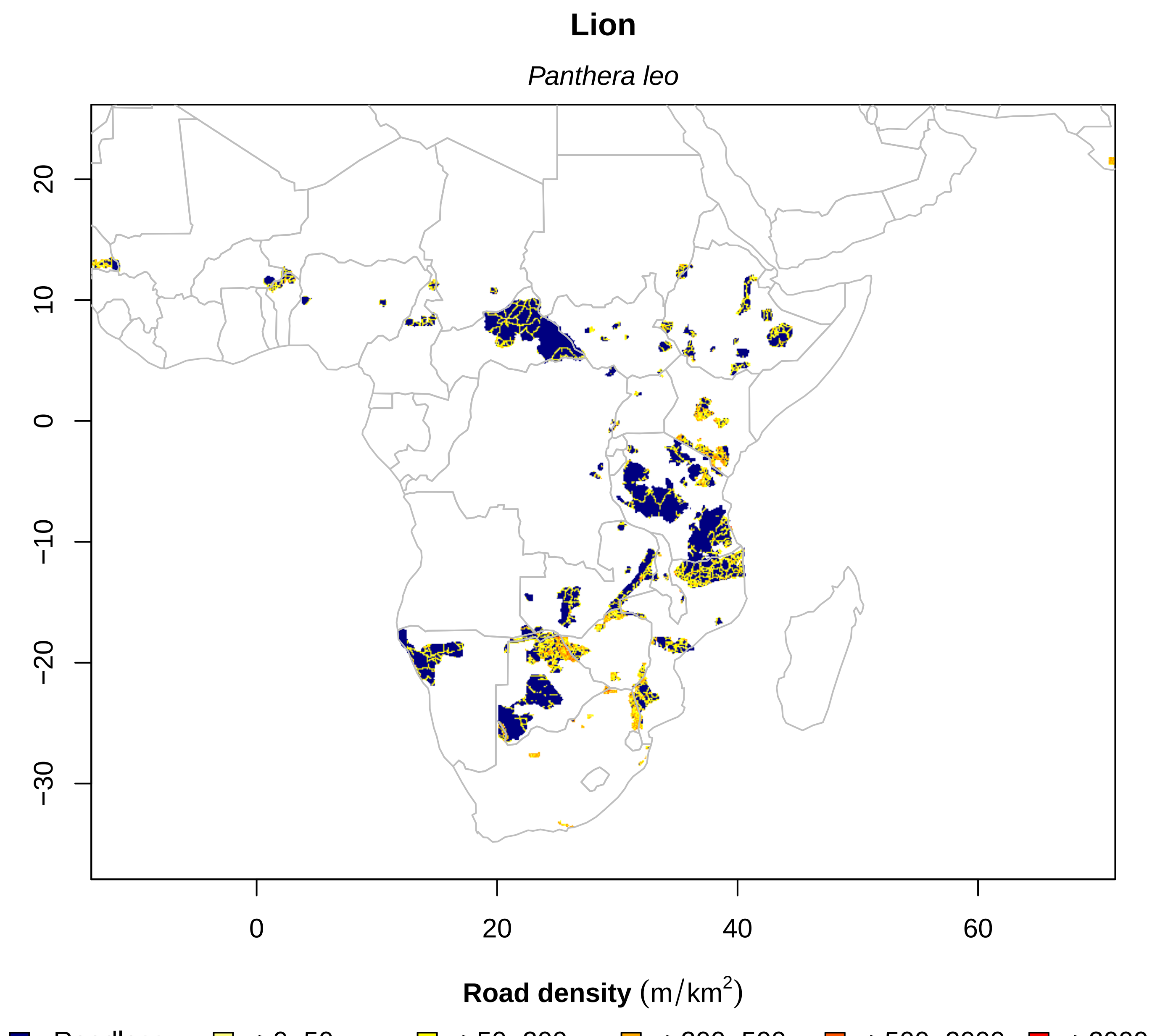

Lion
Panthera leo
20
10
0
−10
−20
−30
0
20
40
60
Road density (m/km²)
Roadless
>0−50
>50−200
>200−500
>500−2000
>2000

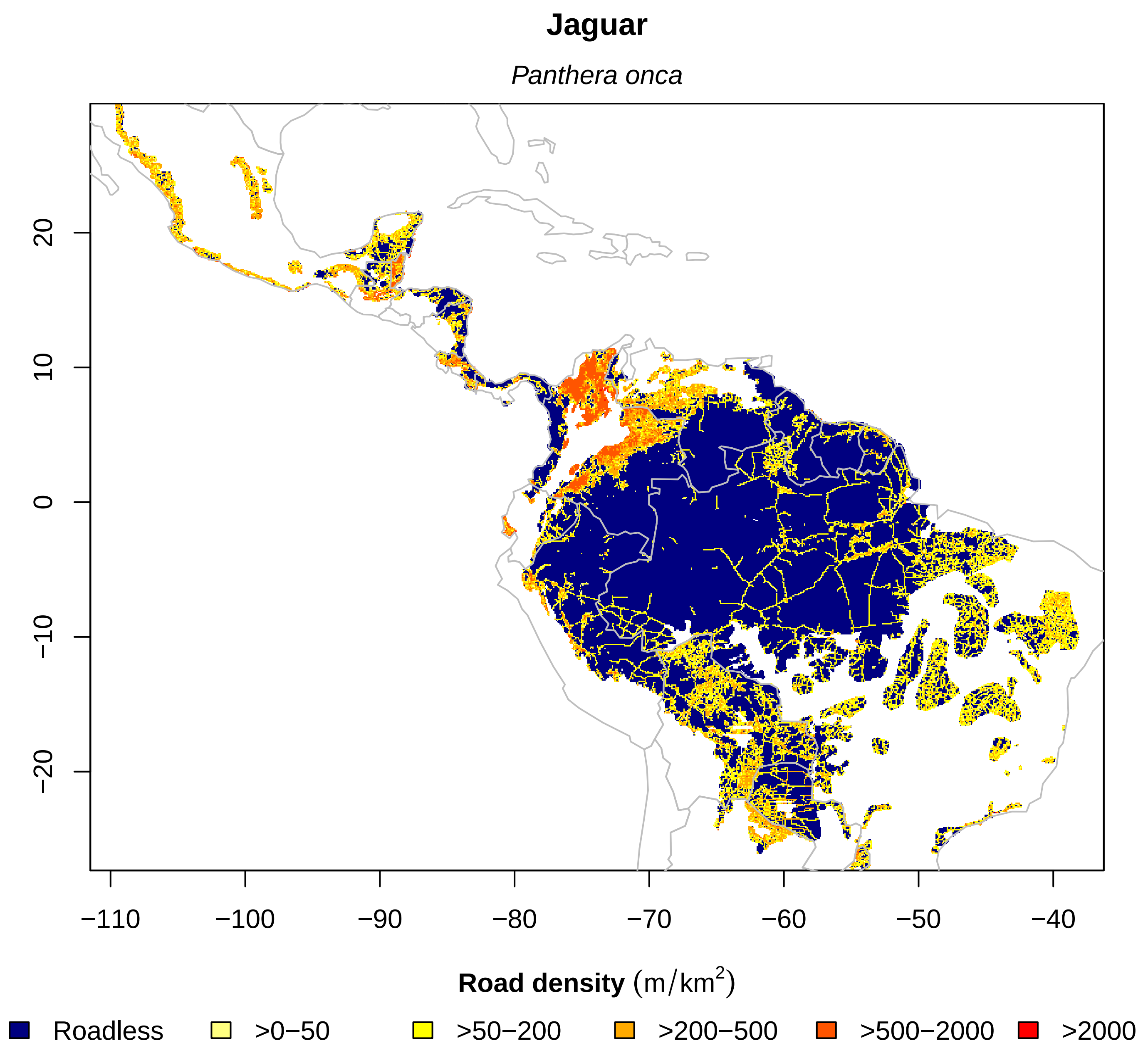

Jaguar
Panthera onca
20
10
0
−10
−20
−110
−100
−90
−80
−70
−60
−50
−40
Road density (m/km2)
Roadless
>0−50
>50−200
>200−500
>500−2000
>2000

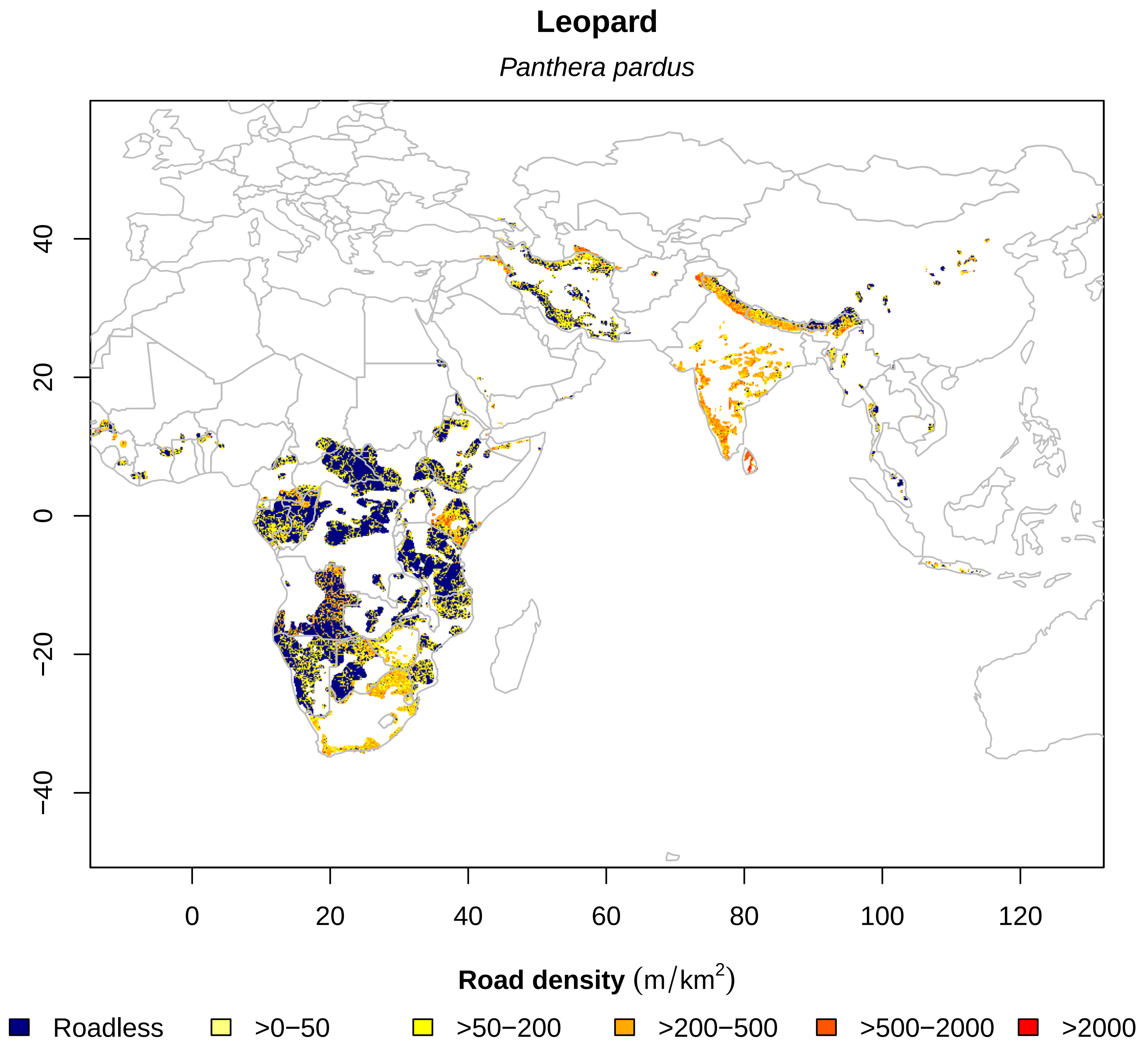
Leopard
Panthera pardus
40
20
0
−20
−40
0
20
40
60
80
100
120
Road density (m/km²)
Roadless
>0−50
>50−200
>200−500
>500−2000
>2000

Tiger

Panthera tigris

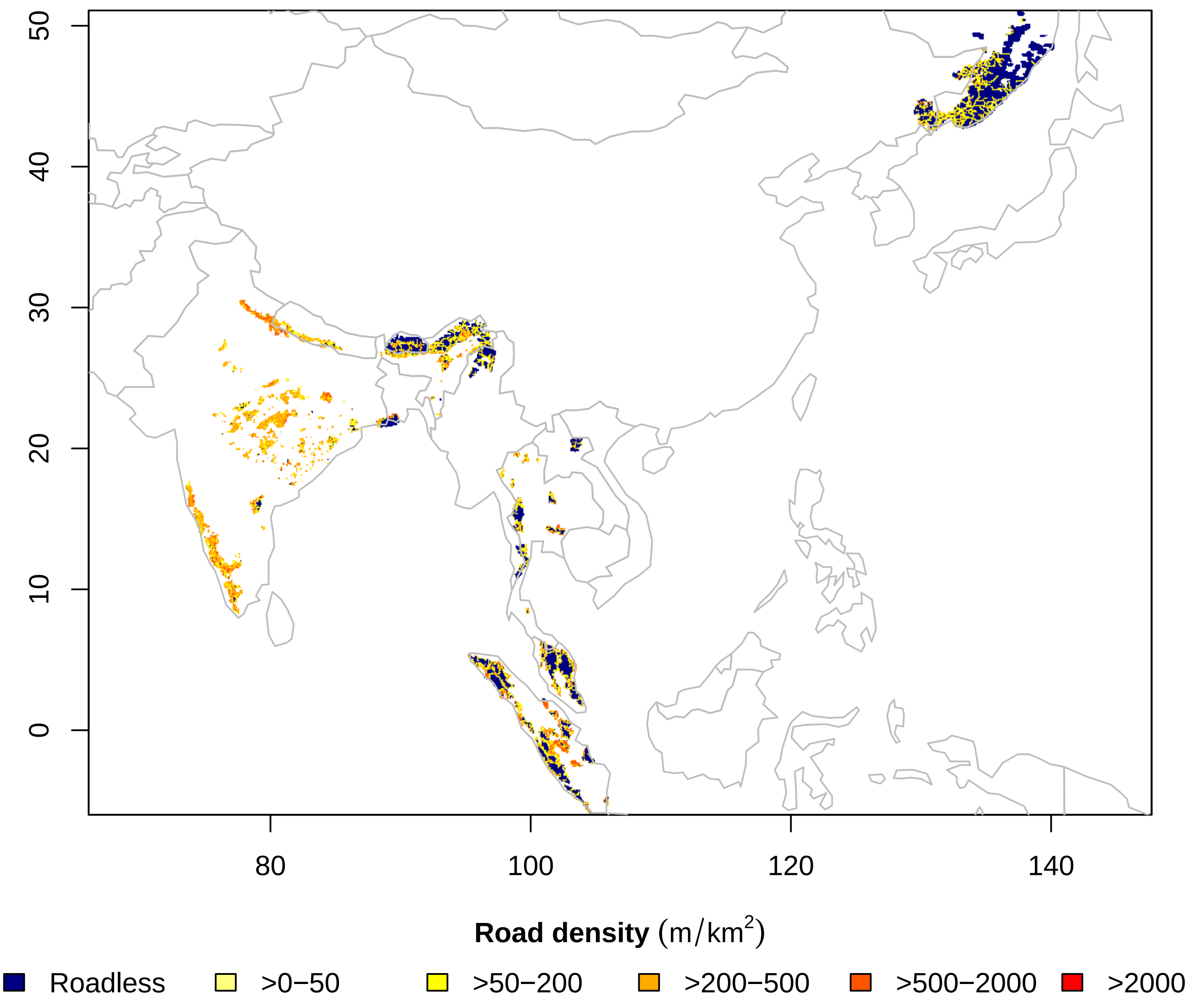

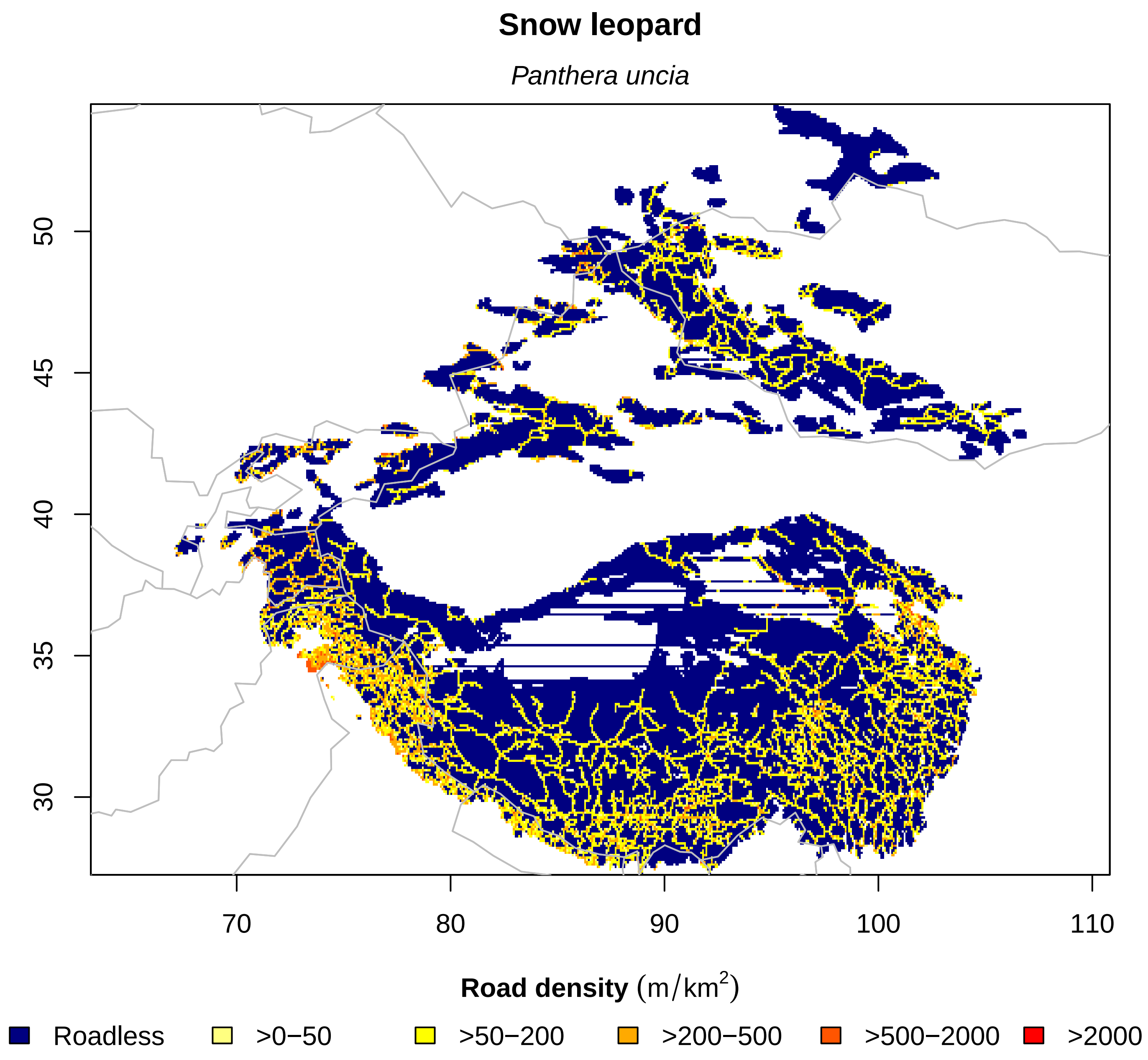

Snow leopard
Panthera uncia
50
45
40
35
30
70
80
90
100
110
Road density (m/km2)
Roadless
>0−50
>50−200
>200−500
>500−2000
>2000

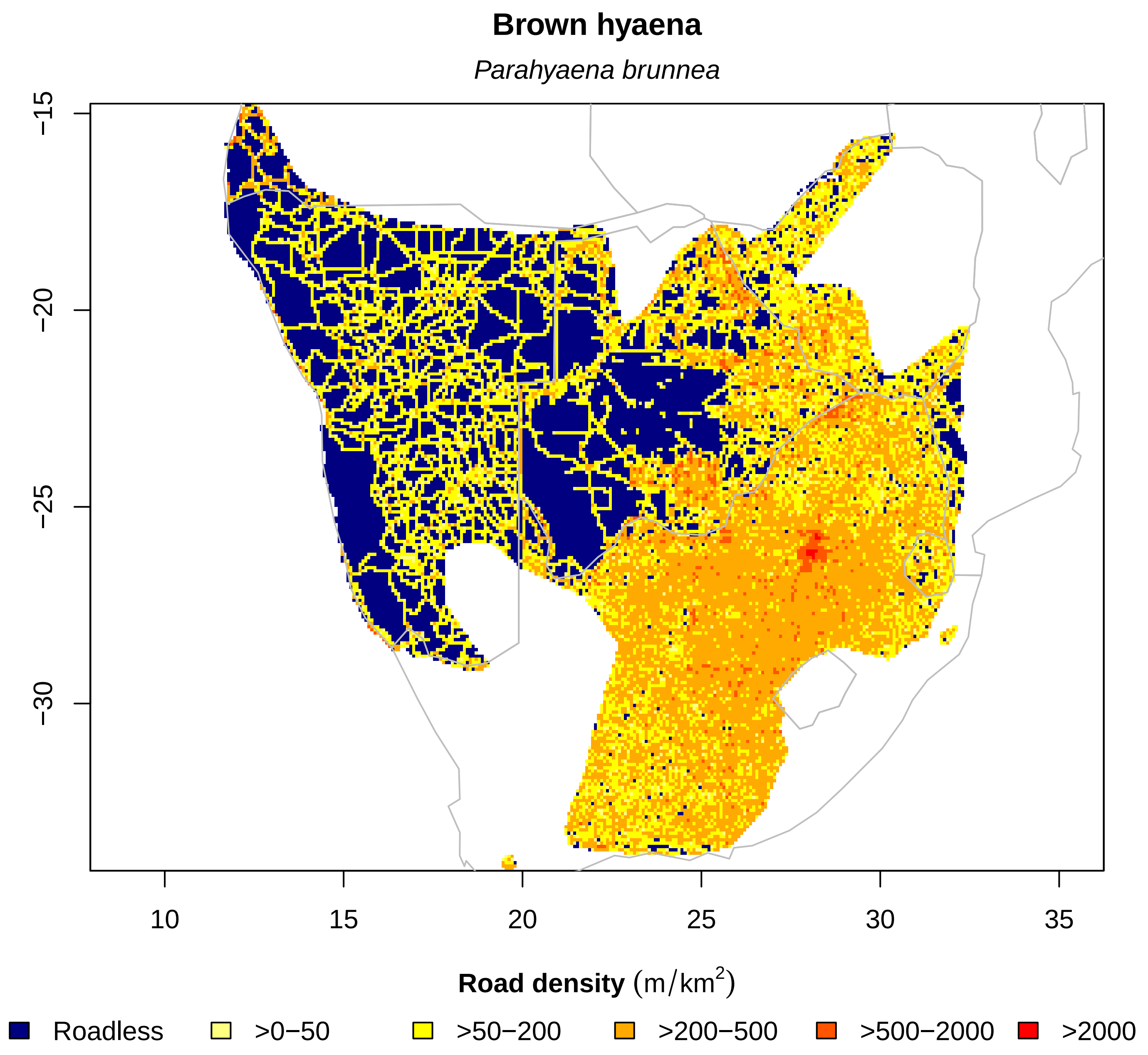
Brown hyaena
Parahyaena brunnea
−15
−20
−25
−30
10
15
20
25
30
35
Road density (m/km²)
Roadless
>0−50
>50−200
>200−500
>500−2000
>2000

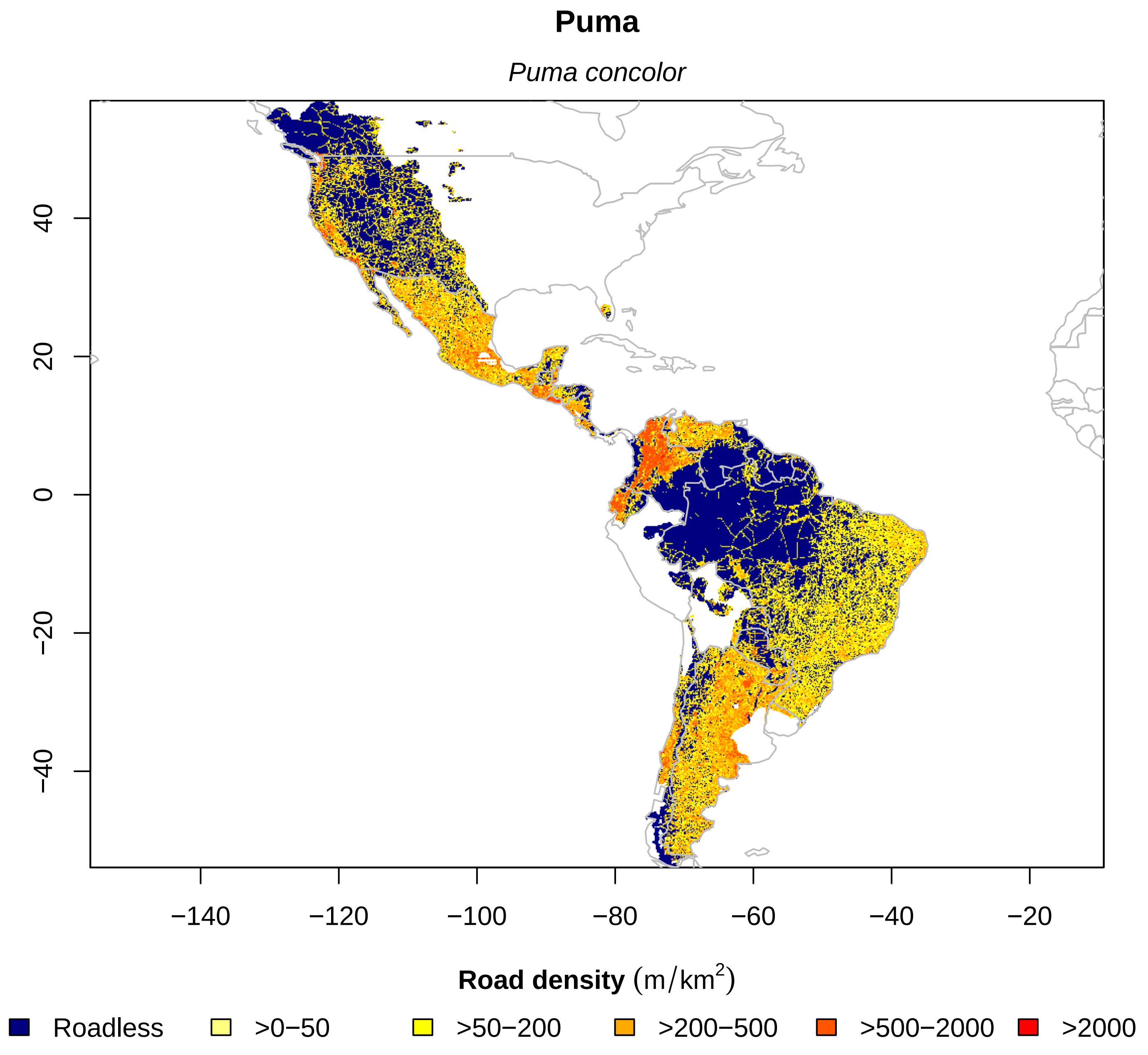

Puma
Puma concolor
40
20
0
−20
−40
−140
−120
−100
−80
−60
−40
−20
Road density (m/km²)
Roadless
>0−50
>50−200
>200−500
>500−2000
>2000

Tasmanian devil

Sarcophilus harrisii

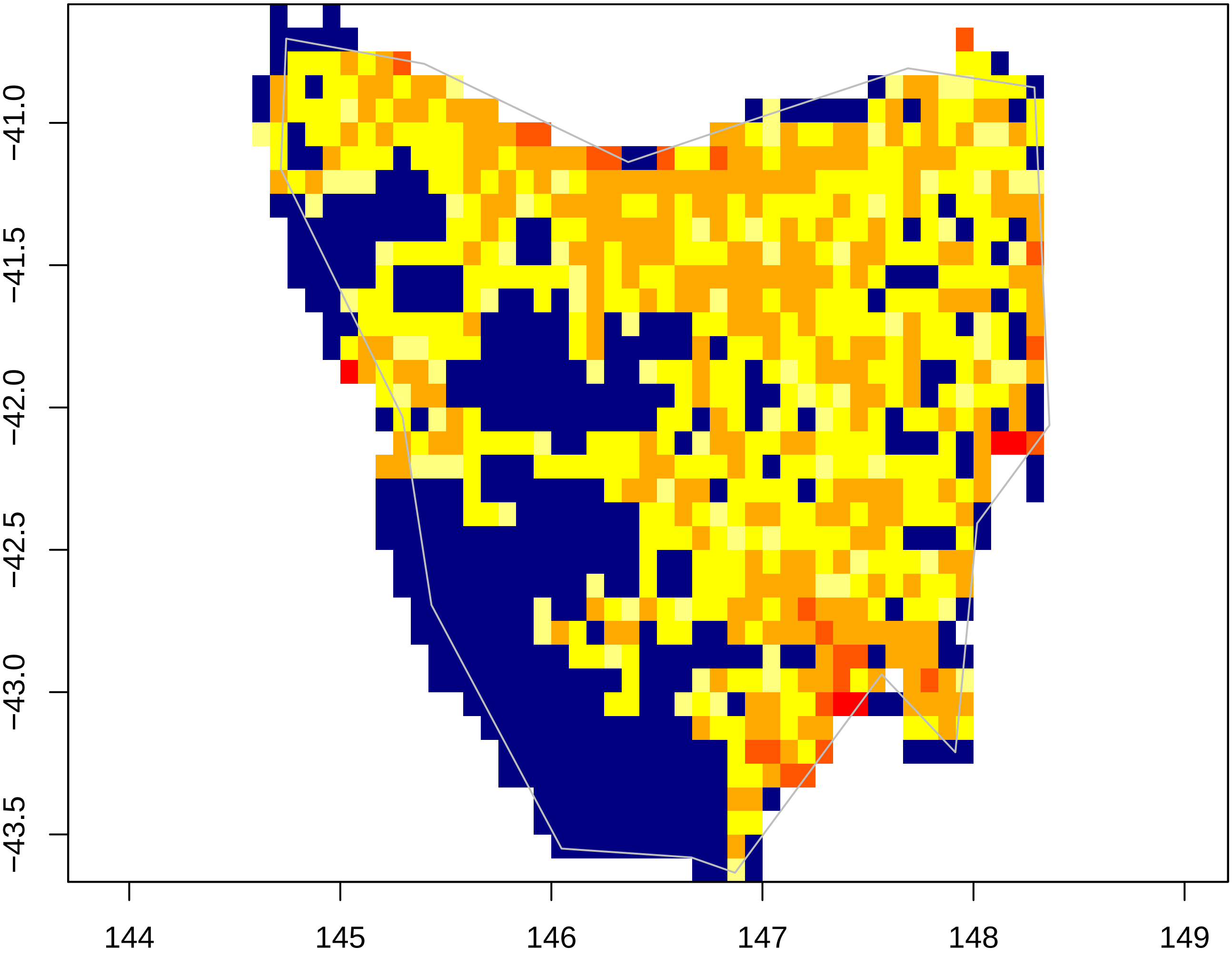

Road density (m/km^2)

Roadless   >0−50   >50−200   >200−500   >500−2000   >2000

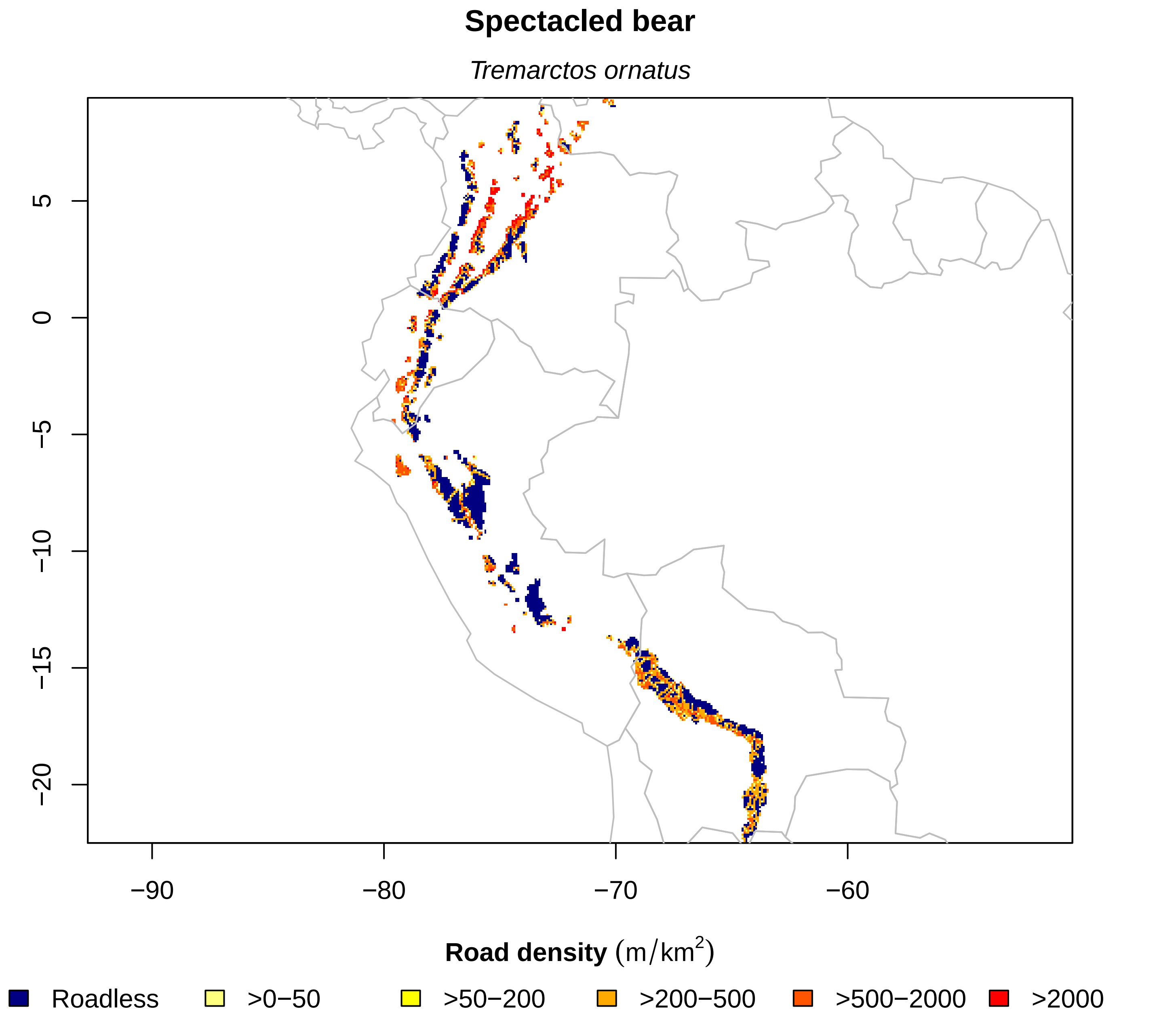
Spectacled bear
Tremarctos ornatus
5
0
−5
−10
−15
−20
−90
−80
−70
−60
Road density (m/km2)
Roadless
>0−50
>50−200
>200−500
>500−2000
>2000

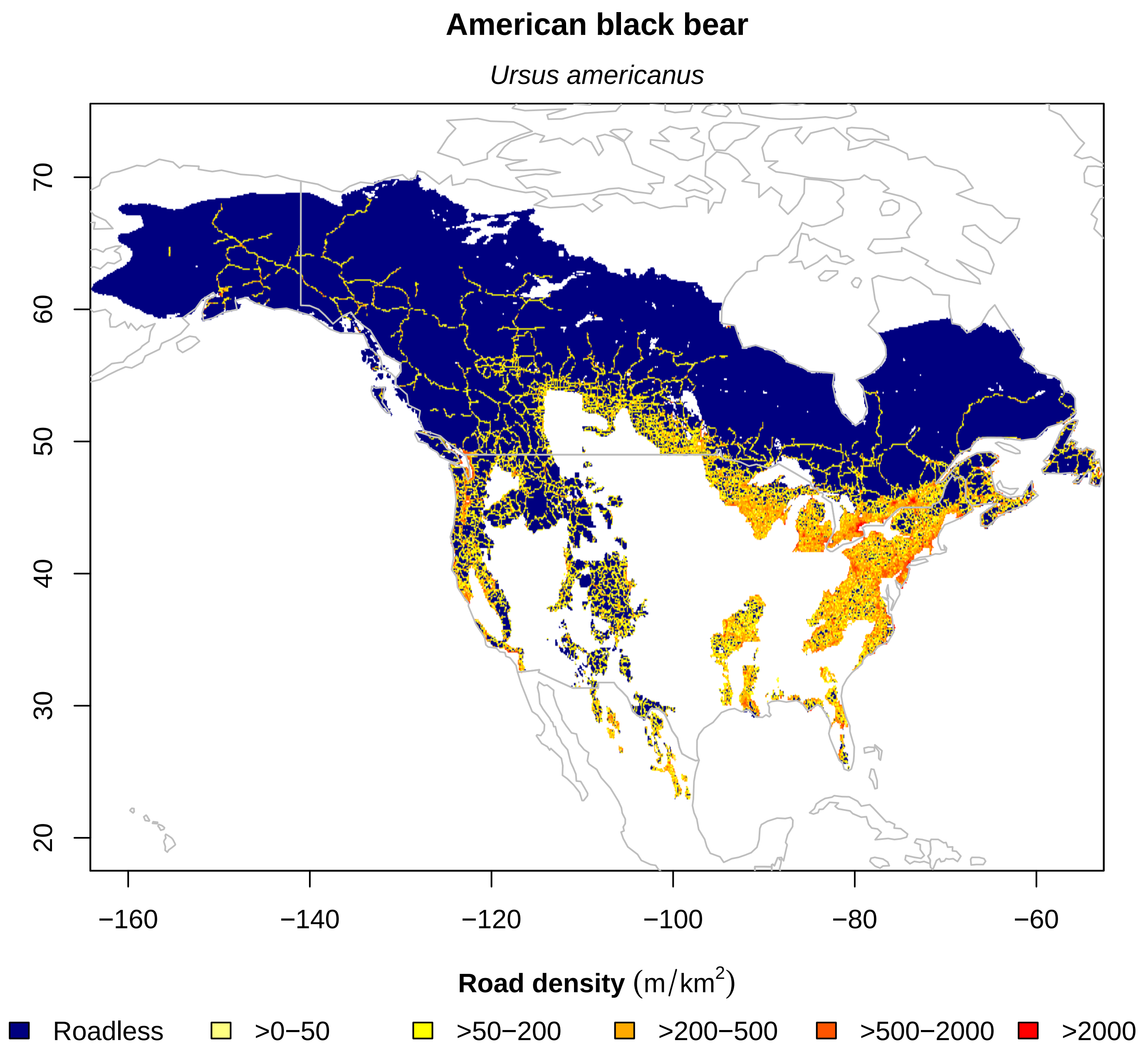

American black bear
Ursus americanus
70
60
50
40
30
20
−160
−140
−120
−100
−80
−60
Road density (m/km²)
Roadless
>0−50
>50−200
>200−500
>500−2000
>2000

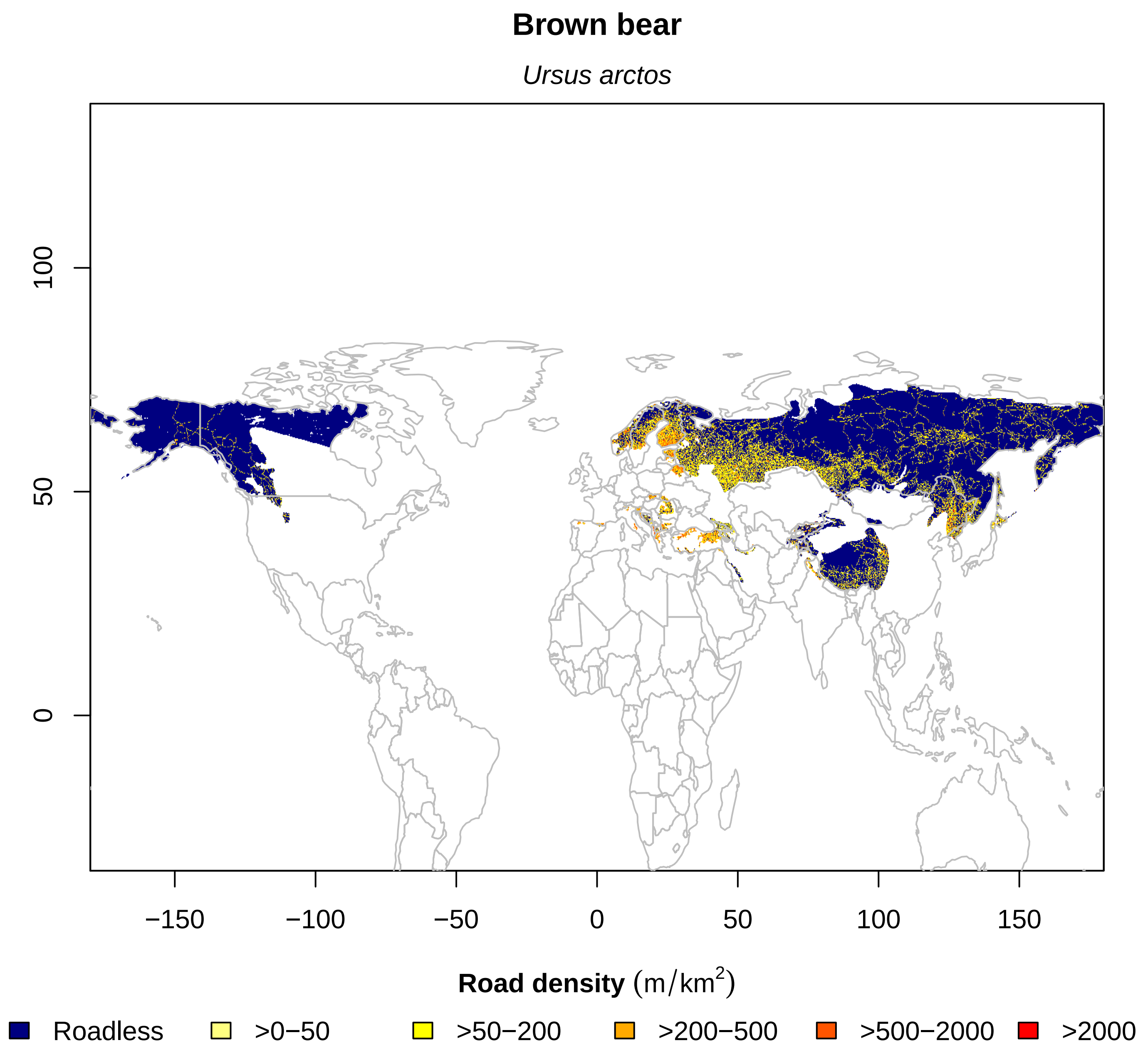
Brown bear
Ursus arctos
100
50
0
−150
−100
−50
0
50
100
150
Road density (m/km2)
Roadless
>0−50
>50−200
>200−500
>500−2000
>2000

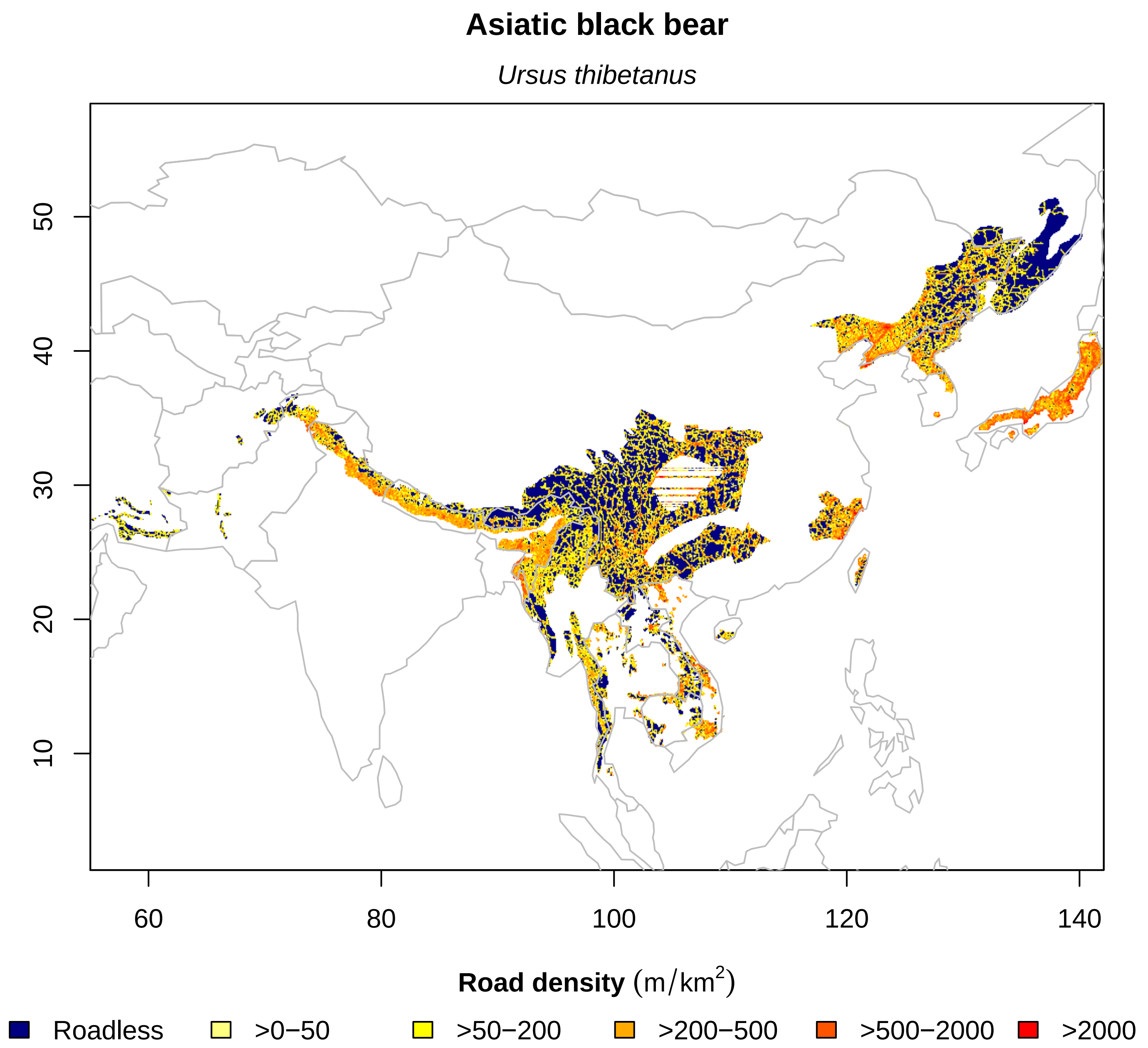

Asiatic black bear
Ursus thibetanus
50
40
30
20
10
60
80
100
120
140
Road density (m/km²)
Roadless
>0−50
>50−200
>200−500
>500−2000
>2000

Supplementary References

1. Wallach, A. D., Bekoff, M., Nelson, M. P. & Ramp, D. Promoting predators and compassionate conservation. *Conserv. Biol.* **29**, 1481–1484 (2015).
2. IUCN. The IUCN Red List of Threatened Species. Version 2018-1. http://www.iucnredlist.org (2018).
3. Jones, K. E. *et al.* PanTHERIA: a species-level database of life history, ecology, and geography of extant and recently extinct mammals. *Ecology* **90**, 2648–2648 (2009).
4. Hollings, T., Jones, M., Mooney, N. & McCallum, H. Trophic cascades following the disease-induced decline of an apex predator, the Tasmanian devil. *Conserv. Biol.* **28**, 63–75 (2014).
5. Wyza, E., Dollar, L. & Stevens, N. First evidence of male spatial associations and roadkill mortality in the fosa Cryptoprocta ferox in Ankarafantsika National Park, Madagascar. *Small Carniv. Conserv.* **56**, 76–85 (2018).
6. Avenant, N. L. *et al.* A conservation assessment of Caracal caracal. *The Red List of Mammals of South Africa, Swaziland and Lesotho, ed. MF Child, D. Raimondo, E. Do Lonh San, L. Roxburgh, and H. Davies-Mostert. South Africa: South African National Biodiversity Institute and Endangered Wildlife Trust* (2016).
7. de Oliveira, T. G. *et al.* Ocelot ecology and its effect on the small-felid guild in the lowland neotropics. *Biology and conservation of wild felids* 559–580 (2010).
8. Krebs, C. J. *et al.* Trophic Dynamics of the Boreal Forests of the Kluane Region. *Arctic* **67**, 71–81 (2014).
9. Jiménez, J. *et al.* Restoring apex predators can reduce mesopredator abundances. *Biological Conservation* vol. 238 108234 (2019).
10. Mora, F. Nation-wide indicators of ecological integrity in Mexico: The status of mammalian apex-predators and their habitat. *Ecol. Indic.* **82**, 94–105 (2017).
11. Meijer, J. R., Huijbregts, M. A. J., Schotten, K. C. G. & Schipper, A. M. Global patterns of current and future road infrastructure. *Environ. Res. Lett.* **13**, 064006 (2018). Data is available at http://www.globio.info
