# Supplementary material for: Severe conservation risks of roads on apex predators
Source: Sci Rep. 2022 Feb 21;12:2902. doi: 10.1038/s41598-022-05294-9 (PMC8861172; doi:10.1038/s41598-022-05294-9)
Supplement: Supplementary file 2 — Supplementary Legends. [file 41598_2022_5294_MOESM2_ESM.pdf]

Description of Additional Supplementary Datasets

Severe conservation risks of roads on apex predators

Quintana I.[1,2,*], Cifuentes E.F.[1,3,4,*], Dunnink J.A.[1,5,*], Ariza M.[1,6], Martínez-Medina D.[1,7], Fantacini F. M.[1,8], Shrestha B.R.[1,9], Richard F-J.[1]

[1]Département de Biologie des Organismes et des Populations, Université de Poitiers, Poitiers, France

[2]Biodiversity and Development Institute, Unit 4, Gunner's Park, Epping 1, Cape Town 7460, South Africa

[3]Department of Plant Sciences, University of Cambridge Conservation Research Institute, The David Attenborough Building, Pembroke Street, Cambridge CB2 3QZ, UK

[4]Laboratorio de Ecología de Bosques Tropicales y Primatología (LEBTYP), Universidad de los Andes, Cra 1 Nº 18A - 12, Bogotá, Colombia

[5]Panthera, 8 West 40th Str. New York, NY, USA. 10018

[6]Natural History Museum, University of Oslo, P.O. Box 1172 Blindern, 0318 Oslo, Norway

[7]Fundación Reserva Natural La Palmita, Centro de Investigación, Grupo de Investigaciones Territoriales para el Uso y Conservación de la Biodiversidad, Bogotá, Colombia

[8]Instituto Ambiental Brüderthal, IAB, 88353-190, Brusque/SC, Brazil

[9]Global Institute for Interdisciplinary Studies (GIIS), P.O. Box 3084, Kathmandu, Nepal

*Corresponding author. Email: itxasoqg@hotmail.com (I.Q.); ed-cifue@uniandes.edu.co (E.F.C.); jeffdunnink@gmail.com (J.D.)

All I.Q., E.F.C., J.D., M.A., D.M.M., F.M.F., B.R.S., F-J.R., conceived, designed, and wrote the manuscript. E.F.C. carried out spatial analysis and maps. I.Q. and E.F.C designed and created formulae and figures. M.A., D.M.M, F.M.F., B.R.S., I.Q., J.D., and F-J.R. collected articles and extracted data for wildlife-vehicle collision.

Supplementary Data 1. Collated wildlife-vehicle collision data ranging from 1963 to 2021. The rate of killed individuals per year was calculated as the fraction of number killed individuals over the number of years.

Supplementary Data 2. Protected areas in the Brazilian Amazon, Africa and Nepal that will be crossed by future road developments and/or affected by a 10-km buffer along the road. Explanation of IUCN category available at: https://www.iucn.org/theme/protected-areas/about/protected-area-categories
